# A New Set of Aromaticity Descriptors Based on the Electron Density Employing the Distributed Multipole Analysis (DMA)

**DOI:** 10.1021/acsomega.4c11451

**Published:** 2025-04-03

**Authors:** Matheus Máximo-Canadas, Roberta Siqueira Soldaini Oliveira, Marco Aurélio Souza Oliveira, Itamar Borges

**Affiliations:** † Departamento de Química, Instituto Militar de Engenharia (IME), Praça General Tibúrcio, 80, Rio de Janeiro, RJ 22290-270, Brazil; ‡ Departamento de Engenharia de Defesa, Instituto Militar de Engenharia (IME), Praça General Tibúrcio, 80, Rio de Janeiro, RJ 22290-270, Brazil

## Abstract

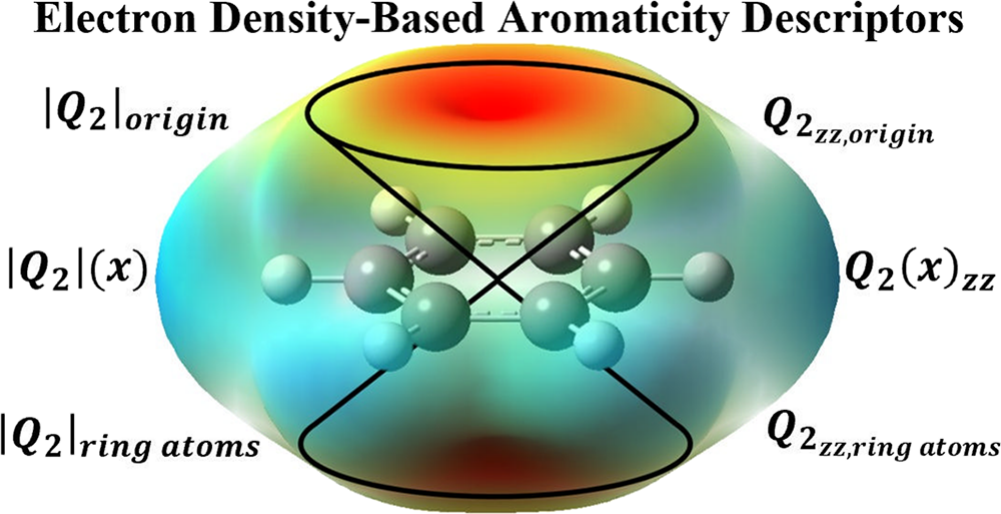

Aromatic compounds are energetically stable and have low reactivity, but an aromaticity concept is difficult to establish because it cannot be measured. For this reason, different descriptors have been developed to rationalize and quantify this phenomenon. Given that cyclic electron delocalization is an essential property of aromatic compounds, in this work, we propose six new aromatic descriptors based on Stone’s distributed multipole analysis (DMA) to partition the molecular electron density into electric multipoles localized on different sites of a molecule. The new aromatic descriptors are based on different components of the DMA quadrupole electric moment tensor ***Q*_2_**, the first term of the DMA multipole expansion having contributions from the out-of-plane electron density. The proposed descriptors are straightforward to obtain because the DMA method is implemented on different popular electronic structure packages. For users of Gaussian, the formatted checkpoint file with the calculated molecular electron density is used as input for the GDMA2 program of Stone to compute the necessary ***Q*_2_** components. A Python script is provided to calculate the proposed descriptors. The computer protocol for determining the ***Q*_2_**-based descriptors in either way is presented. To assess the performance of the aromaticity descriptors, we used 12 tests of the Girona benchmark developed by the Solà group involving different distortions of benzene, substitutions, complexation, ring size dependence, atom size dependence, heteroatomic species, Clar systems, and fulvenes. The descriptors normalized by the corresponding benzene values presented the most consistent results. The correct aromaticity trends of the six normalized new indices were predicted entirely in 65% of the cases; for 22%, most trends were predicted and only failed utterly in 13%. The failed cases were related to the molecular symmetry, which led to the partial cancellation of the ***Q*_2_** tensor components, thereby affecting the aromatic descriptors and, in some cases, due to a contamination of sigma electrons. Our proposal joins others to contribute to understanding the important and complex chemical concept of aromaticity.

## Introduction

Aromaticity is a crucial and ubiquitous concept in chemistry that cannot be measured in a laboratory. The beginning of a more accurate understanding of the subject can be traced to mid-19th century when Kekulé suggested the possible structure of the benzene molecule, the now universally recognized hexagonal structure of carbon atoms with alternating single and double bonds.^1^ Along with this proposal, other nonobservable concepts, such as resonance, electronic delocalization, and atomic orbitals, became highly relevant to chemistry.^2−6^

Based on Kekulé’s proposal, benzene has been considered the leading and most crucial aromatic center of organic molecules. A thorough understanding of the aromaticity phenomenon is paramount, given the many existing aromatic compounds and those yet to be discovered. Therefore, there is a great interest in understanding and rationalizing aromaticity.^7−11^

Aromatic molecules are crucial in various fields, from organic synthesis to materials science.^12,13^ These molecules are essential in numerous applications, including pharmaceuticals, electronic materials, and catalysts. The importance of the aromaticity concept is evidenced by its ability to rationalize the reactivity and stability of compounds, thus contributing to the prediction and design of new molecular systems. The continuous evolution of criteria and methods for evaluating aromaticity by employing indices (used here interchangeably with the word descriptor) based on electronic delocalization and electronic structure methods, especially density functional theory (DFT), reflects the complexity and relevance of this concept in modern chemistry.^14^

**Figure 1:**
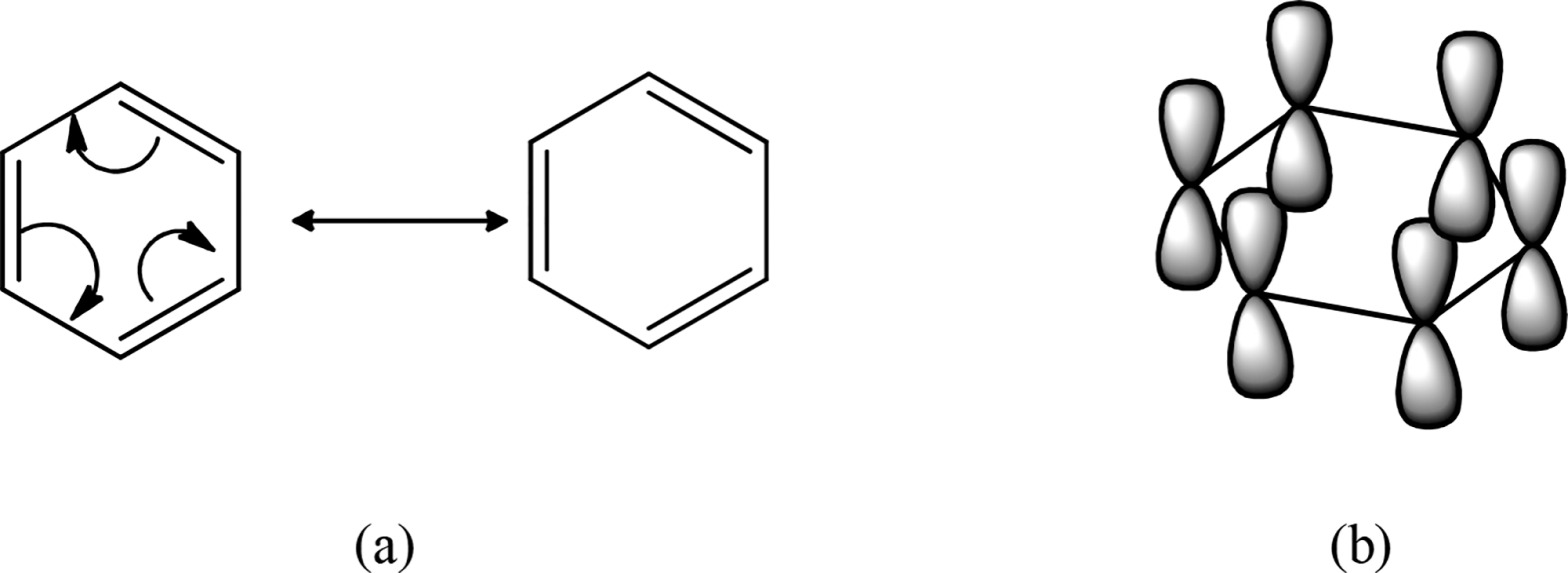
(a) Resonance structures of benzene showing its electronic delocalization; (b) *p*-orbitals and π-electron density in benzene aromatic ring.

According to Kekulé’s model, observations stemming from the benzene molecule allow one to consider some typical characteristics of aromatic compounds. Since benzene is a cyclic molecule with six carbon atoms alternating between saturated and unsaturated bonds, one might logically imagine it a deformed hexagon. However, this is not observed because benzene is a regular, perfectly symmetric hexagon with bonds of the same length in a planar configuration. This is a consequence of the resonance state existing between hybrid theoretical structures with alternating bonds between carbon atoms in a permanent state of electronic delocalization,^15,16^ as illustrated in Figure 1a. A commonly accepted representation of this aromatic structure shows π bonds bearing electron density distributed above and below the plane of the ring, freely transitioning within the empty *p* orbitals perpendicular to this plane and overlapping with each other. This model best represents the electronic density within the aromatic ring (Figure 1b).

Faraday had already isolated benzene in the early 19th century, and even then, its low reactivity was observed, leading to the idea of the compound’s energetic stability.^17^ This characteristic behavior is found in this precursor to aromatic molecules and many others, including inorganic and organometallic compounds, which began to be synthesized in the 20th century.^18,19^ However, although the aromaticity phenomenon is well characterized today, its origin and a complete understanding are still not fully established. Multiple factors must be considered to describe it comprehensively because it is a multifaceted phenomenon.^9,20−22^

Aromaticity cannot be quantified directly because it is not an observable. However, the measurement of related properties can be quantified.^18,19,23^ This quantification can be achieved by measuring specific properties directly related to or associated with the aromaticity phenomenon, such as (i) the equalization of bond lengths – all bonds in an aromatic system, regardless of their saturation, have the same length^16^; (ii) energetic stability – aromatic molecules are more stable than others that are structurally similar^14,17^ and; (iii) magnetic behavior.^18,24,25^

The aromaticity indices are the best way to conceptualize aromaticity quantitatively. The most accurate approaches in this regard are based on electronic delocalization. Descriptors such as the Aromatic Fluctuation Index (FLU), the Para-Delocalization Index (PDI), and the Multicenter Delocalization Index (MCI) have proven to be highly sensitive to small changes in aromaticity.^13,26^ These indices assess the degree of electron sharing between contiguous atoms and the uniformity of this sharing, which is significant in aromatic molecules. Methodologies based on the Electron Localization Function (ELF) have further refined this approach by separating the σ and π components.^27,28^

In addition to electronic criteria, indices based on magnetic properties, such as the strength of ring currents induced by an external magnetic field computed by NICS-based (Nucleus Independent Chemical Shifts) indices, are also widely used.^22,24,25,29^ Energetic criteria, such as the energetic stabilization associated with aromaticity, are also considered, although they may present limitations in specific contexts.^8,30^

It is incorrect to reduce aromaticity simply to electronic delocalization with its high electron density of π-electrons out of the plane of the ring in the bonds of a molecule. However, this factor is likely the main one.^9,19^ Moreover, electronic delocalization is not a directly measurable parameter.^14,16^ Nevertheless, considering the intimate relation between electronic delocalization and aromaticity, descriptors based on the former to quantify the latter can be very useful.^9^ Since electron density is a physical observable, it can be determined by techniques such as electron diffraction. Additionally, it is a concept with a solid theoretical basis in the framework of DFT.^31^ These are two important reasons for attempting to define an aromaticity descriptor based on the electronic density.

Methods for quantifying electronic delocalization in aromatic systems are varied and rely on different structural, electronic, and magnetic properties. A recent method is the Electron Density of Delocalized Bonds (EDDB), which measures electron delocalization and has been shown to effectively predict and quantify the relationship between bond length alternation, electronic delocalization, and diatropicity – three criteria for evaluating aromatic systems. This makes EDDB an efficient method for measuring aromaticity. Diatropicity is a property associated with aromatic systems, which exhibit a characteristic ring current due to delocalized electrons, leading to an induced magnetic field that affects the surrounding chemical environment.^32,33^ Vibrational spectroscopy is also employed for examining aromaticity by using local stretching force constants of vibrational modes to derive an Aromaticity Index (AI) that quantifies π-delocalization in conjugated cyclic systems.^34^ Three-dimensional isotropic magnetic shielding (IMS) maps allow visualization of electronic delocalization in twisted polycyclic aromatic hydrocarbons, offering a detailed and quantitative assessment of aromatic character.^35^

Other indices, such as the aforementioned PDI and FLU, are based on the quantum theory of atoms in molecules and measure electronic delocalization between related atoms in six-membered rings.^36,37^ The analysis of the induced magnetic field is also a useful tool for estimating electronic delocalization and aromaticity, particularly in planar systems using, for instance, NICS.^25,38^ These methods demonstrate that indicators based on electronic delocalization show high sensitivity to subtle changes in aromaticity, encouraging the use of multiple indicators for a comprehensive understanding of the phenomenon.^13^

Since aromatic compounds are energetically stable and have low reactivity, if there is any possibility of geometric, conformational, or electronic adjustment within a molecule that could render it aromatic, the molecule will adopt such a configuration. This property has implications for the chemical reactivity of the molecules. For example, aromatic compounds strongly favor substitution reactions in their rings – both electrophilic and nucleophilic aromatic substitutions, and unlikely, or rarely, undergo electrophilic addition reactions to their electron density. Otherwise, this would extinguish the electronic delocalization, thus ceasing the aromatic character. All of this is for maintaining the integrity of the aromatic structure.^14,16^

Despite its unsaturation, benzene’s measured C–C bond length has a unique value, intermediate between a double bond and a single bond equal to 1.39 Å. This is due, as mentioned, to the resonance structure of the benzene ring resulting from the electronic delocalization (Figure 1a). However, not every molecule has this bond length equalization in conjugated cyclic systems. In other words, electron delocalization is not a general phenomenon for all cyclic molecules of this type. Krygowski and collaborators proposed a mathematical model called HOMA (Harmonic Oscillator Model of Aromaticity), which quantifies the relationship between bond length and electronic delocalization.^39^ The HOMA index ultimately describes the decrease in the aromaticity of a system. Therefore, the lower the aromaticity of the compound, the lower the HOMA index, with an ideal maximum value of 1. Useful variants of the HOMA descriptor have been developed.^40^

Krygowski and his group, using the HOMA index, discovered that the aromaticity of a π-electron system decreases when two structural properties are modified: (i) the alternation of bond lengths increases and; (ii) the average bond lengths elongate.^41^ Schleyer et al. also found, using NICS, the importance of bond length equalization, which is associated with π-electron delocalization and, therefore, a more prominent aromatic character.^22,25^ Soncini et al. discovered through π-current density maps that when using only saturated clamping groups (i.e., groups attached to a benzene ring that do not significantly alter the ring’s electronic properties), the benzene ring’s current remains essentially unchanged.^42^ Therefore, the benzene molecule can afford substantial alternations in C–C bond lengths without significantly losing its delocalized cyclic electron density and, thus, its aromaticity.^43,44^

An excellent and up-to-date analysis and presentation of the aromaticity phenomenon and its various descriptors can be found in the book by Solà and coauthors.^9^ Several of its chapters are cited in this text. Applications of different aromaticity to different systems indices abound in the literature.^13,45^

The proposal of aromatic indices implies that the choice of theoretical methods can significantly influence the results, as observed in benchmark studies of different aromaticity indices.^14,23,46,47^ The necessity of multiple indicators for a comprehensive understanding of aromaticity is also a point of debate, as different indices may provide complementary information.^12,13^

Among all possible quantifiable parameters (indices) that can be used to describe aromaticity, the analysis of delocalized electron density is the focus of this work, as it provides a good characterization of the aromaticity state of a molecule. In this work, we explore a widely used method, the Distributed Multipole Analysis (DMA) of Stone,^48−51^ to partition the molecular electron density into electric multipoles distributed throughout the molecule to propose new aromaticity descriptors. We have been exploring the DMA to rationalize a variety of different chemical phenomena, including the sensitivity of energetic materials^52−59^ and the rationalization of hydrodesulfurization (HDS) catalytic processes.^60−63^ In this work, to test the DMA-based proposed aromatic descriptors, we employ 12 test sets of aromatic and antiaromatic molecules from the Girona benchmark proposed by the Solá group.^18,19^

## Theoretical and Computational Methods

### Distributed Multipole Analysis (DMA) and the Chemical Interpretation of its Electric Multipole Terms

The computation of multipolar electric moments at convenient locations in a molecule is a direct and compact way to describe the electron density distribution across a molecule.^50^ The electric multipolar moments can be mathematically defined as either Cartesian or spherical tensors, which provide a compact way of expressing physical quantities independently of the coordinate system. Spherical tensors are based on spherical harmonics, and the Cartesian and spherical descriptions are directly related.

For a molecular electron density calculated using any electronic structure method (i.e., employing DFT or an ab initio method), DMA describes it as an expansion of electric multipoles located at the atoms or any other convenient point within or in the neighborhood of the molecule. The electron density is the sum of products of Gaussian functions centered on atoms, with coefficients determined from the one-electron density matrix. For orbitals on different atoms, each pair of Gaussian functions produces a finite multipolar series at a point between the two atoms; the exponents of the corresponding Gaussian orbitals determine this point. These multipoles are represented as a series at different expansion sites. DMA evaluates these exact representations, and each is approximated by a rapidly converging multipolar expansion centered on different locations of the molecule. Combining the electron density with the positive charges of the nuclei produces the molecular charge density.^48−51^

The DMA multipoles have a straight chemical interpretation. The product of two “*s*” functions is spherically symmetric and corresponds to a point charge (a monopole located at the atomic site), which is a rank-0 tensor represented by ***Q*_0_**. The product of an “*s*” function with a “*p*” function represents both a charge and a dipole, the latter being a rank-1 tensor ***Q*_1_** that indicates local polarization effects**_._** The overlap of two “*p*” functions represents a charge (***Q*_0_**), dipole (***Q*_1_**), quadrupole (a rank-2 tensor ***Q*_2_**), and so on.^50^ Any individual product of the atomic-centered basis functions is expanded as a sum of electric multipole moments up to the degree of its polynomial. Although the DMA expansion can be extended to higher orders (e.g., octupole), for our purposes, we stop the expansion in the quadrupole term: the first three multipole terms provide an accurate picture of the electron density of a molecule. Therefore, the DMA partitioning into electric multipoles at different molecular sites provides a detailed, accurate, and chemically intuitive picture of the charge density.

The quadrupole ***Q*_2_** term is important for our purposes here because it is the first multipole term in the DMA expansion to include contributions from the electron density outside the molecular plane and, thus, is associated with delocalized π-electrons, such as those in unrestricted electron pairs (an electron pair in which the up and down spins are not restricted to share the same spatial orbital) or those involved in unsaturated bonds. Therefore, our proposal here is to use different components, or combinations of them, of the ***Q*_2_** DMA tensor components to quantify the electron delocalization in a molecule or its neighborhood to propose descriptors to assess the aromaticity quantitatively.

### DMA Quadrupole Moment Tensor (***Q*_2_**)

The quadrupole moment tensor ***Q*_2_** describes a quadrupolar charge distribution corresponding to two positive and two negative charges. The quadrupole moment is the first electric moment to include contributions from the out-of-plane electron density.^64^ It is used in this work to propose new descriptors to quantify aromaticity.

**Figure 2:**
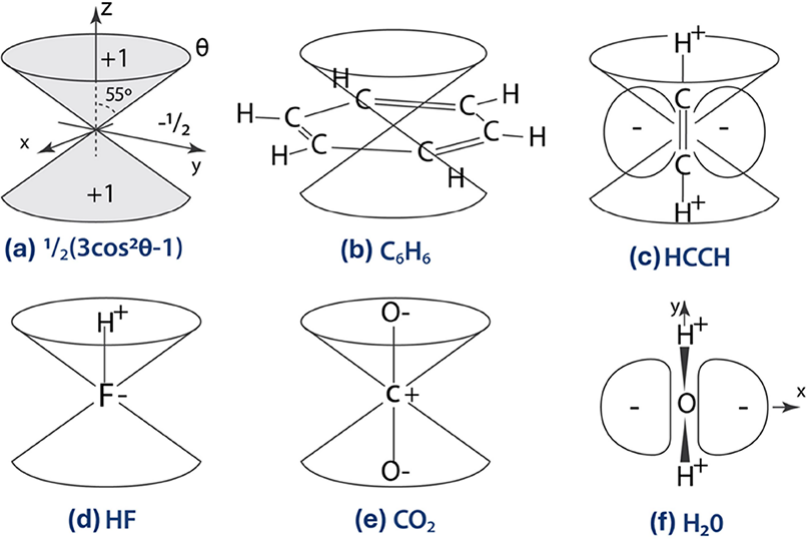
Quadrupole moments. In panel (a),  is the angular part of Θ_*zz*_. Adapted with permission from *The Theory of Intermolecular Forces*, 2013. Copyright 2013 by the Oxford University Press.

The ***Q*_2_** tensor has the following components:
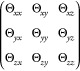


given in atomic units (*ea*_0_^2^).The Θ*_*zz*_* component, for instance, is defined as
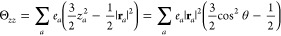
1where in the last term, the vector **r**_*a*_ is expressed in spherical polar coordinates, *x*_*a*_, *y*_*a*_ and *y*_*a*_ are the Cartesian components of **r***_a_*, and *e*_*a*_ is the electric charge localized at **r**_*a*_. The expectation value of the quadrupole moment for a particular state, for instance, for the Θ*_*zz*_* component, is given by
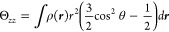
2where ρ(***r***) is the molecular *charge* density. The angular factor in parentheses in eq 2 is positive when θ < 54.7° or θ > 125.3°, and is negative between these values in the region of *xy* plane – see Figure 2a (Figure 2 is adapted from Stone^50^). For instance, for CO_2_ (Figure 2e), the negatively charged oxygen atoms are in regions where the angular factor is positive. In contrast, the positively charged carbon atom occupies the region near the origin (i.e., small *r*), so it contributes little to the quadrupole moment.^50^ Therefore, we expect a negative quadrupole moment, which the measured value (−3.3 *ea*_0_^2^) confirms.^65^

The quadrupole moment tensor has other Cartesian components besides Θ*_*zz*_*, which are given by
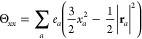

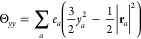

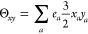

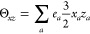

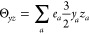


The components of the quadrupole moment tensor ***Q*_2_** are proportional to the spherical harmonics of rank 2, hence the number of independent components is five, the same number of spherical harmonics of rank 2 (a tensor of rank *n* has 2*n* + 1 independent components). In the case of the Θ*_*xx*_* and Θ*_*yy*_* components, it is more convenient to consider Θ*_*xx*_*–Θ*_*yy*_* instead of the separate quantities.

The quadrupole multipole moment is defined from the regular spherical harmonics *R***_2*m*_**(***r***) as
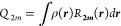
3

For practical applications, it is more convenient to use the real form:
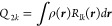
4where *k* is a label for ***Q*_2_** that denotes a member of the series 0, 1*c*, 1*s*, 2*c*, 2*s*,... For the quadrupole tensor, the relation between the spherical and Cartesian representations is given by
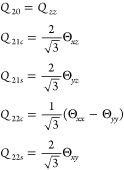
5

We are going to use the spherical representation of the ***Q*_2_** components. Determining the inverse relationships between the Cartesian representation and the above spherical representation is straightforward.^50^

Aromatic indices based on the elements of the ***Q*_2_** quadrupole tensor components can be computed for an entire molecule, any cyclic or noncyclic π-electron neutral and charged systems, and a fragment. Hence, we can have ***Q*_2_-**based global and local aromaticity indices.

Concerning charged species, given that DMA is based on expanding the total electron density into a multipole series, the presence of a net charge in a molecule does not compromise its validity or effectiveness. For instance, in the test T8 of the aromaticity Girona benchmark (see below), we analyzed two charged molecules, namely, C_7_H_7_^+^ and C_8_H_8_^+2^. For both cases, the DMA quadrupole moment components could be computed successfully.

### New Aromaticity Descriptors Based on Spherical Components of the ***Q*_2_** DMA Quadrupole Tensor

Most molecules can have their frameworks (i.e., their rings) on the *xy* plane, which is a convenient and arbitrary choice. Therefore, an obvious aromatic descriptor is based on the *total sum* for a molecule of the out-of-plane quadrupole tensor components *Q*_20_ = *Q*_*zz*_ of *all* atoms, computed at the origin of the molecule, ***r*_origin_** = (0, 0, 0):
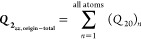
6

In the DMA framework implemented in the GDMA2 program of Stone (see below), multipole moments are computed at both atomic sites and the origin of the coordinate system by default; however, GDMA2 also allows for user-defined expansion points. The software assigns the origin automatically at (0, 0, 0) in the chosen coordinate system. This typically coincides with the geometric center of a molecule (such as the center of a ring), but it is not always the case. Users can specify a different origin for calculating the multipole moments, providing greater flexibility in the analysis. In other words, the quadrupole moment tensor (and the other multipole moments) at the origin or any additional points is inherently origin-dependent and must be checked carefully in each case.

A descriptor more consistent with the idea of aromaticity as a manifestation of electron delocalization of ring atoms is obtained by summing up only the *Q*_20_ = *Q*_*zz*_ or the |***Q***_**2**_| values (see definition of |***Q*_2_**| in eq 7 below) of each ring *carbon* atom, including eventual noncarbon atoms in the case of heterocyclic rings. We name these two indices ***Q***_**2**_*z***z,ring atoms**__ and |***Q***_**2**_|**_ring atoms_**, respectively.

The magnitude of the ***Q*_2_** multipole moment at some point in the molecule is defined as
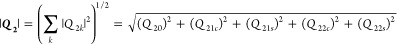
7

We define |***Q***_**2**_|**_origin-total_**, similarly to ***Q***_**2**_***zz*****,origin-total**__ in eq 6, as the index computed for *all* atoms with respect to the origin of the coordinate frame of a molecule at ***r***_origin_ = (0, 0, 0), which is independent of the axis system.^50,64^ Naturally, this index can be “contaminated” by non-π (i.e., σ) in-plane contributions of the electron density, so it works only when the σ contributions given by the spherical tensor elements having *x* and *y* components, namely, *Q*_21*c*_, *Q*_21*s*_, *Q*_22*c*_, and *Q*_22*s*_ (see eqs 5) are not appreciable.

**Figure 3:**
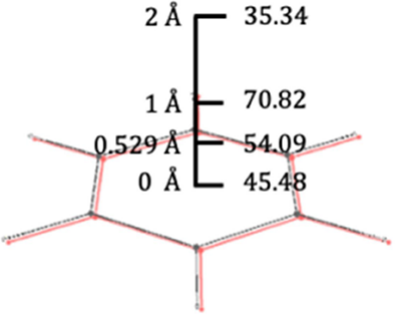
Benzene structure and its respective electron density values at different distances from the aromatic ring plane in *ea*_0_^2^.

One alternative to avoid a possible “contamination” from the *x* and *y****Q*_2_** quadrupole terms (i.e., from the σ system) to an aromaticity descriptor is to compute it at the same arbitrary distance over or below the aromatic ring. Soncini and co-workers suggested that 1 Bohr radius (*a*_0_ = 0.529 Å) above the ring lies near the maximum of the π charge and current densities.^42^ To find the closest distance to this maximum of the electron density, we computed for benzene the *Q*_20_ = *Q*_*zz*_ values at the coordinates ***r*** = (0, 0, *z*) for *z* = 0, 0.5, 0.529 Å (= *a*_0_), 1, 1.5, 2, 2.5, 3, 3.5, 4, and 4.5 Å – see these *Q*_*zz*_ values depicted in Figure 3 and in the more detailed Figure S1 of the Supporting Information. Considering that *z* = 1 Å gives the largest *Q*_*zz*_ value over the center of the benzene ring, hence we define the aromatic descriptor *Q*_**2**_(**1**)_**zz**_ based on it. We also define the corresponding index for the magnitude of the ***Q*_2_** multipole moment computed at the same position, (0, 0, 1 Å), |***Q***_**2**_|(**1**). The six proposed aromatic descriptors are depicted in Table 1. Note that *Q*_22*c*_ and *Q*_22*s*_ components of the ***Q*_2_** tensor in eqs 5 have only the Cartesian *x* and *y* contributions. Therefore, their values indicate contributions from sigma bonds in a *Q*_**2**_**-**based descriptor that includes these components.

**Table 1:** Proposed Six Aromaticity Descriptors Based on the ***Q*_2_** DMA Tensor Components^a^

aromaticity index	definition
|***Q*_**2**_**|**_ring atoms_**	The total sum of the |***Q***_**2**_| values of each ring atom
***Q***_**2**_**zz,ring atoms**__	The total sum of the *Q*_20_ = *Q*_*zz*_ values of each ring atom
|***Q*_**2**_**|**_origin_**	The total sum of the |***Q***_**2**_| values computed for all the atoms of a molecule referred to ***r***_origin_ = (0, 0, 0)
***Q***_**2**_**zz,origin**__	The total sum of the *Q*_20_ = *Q*_*zz*_ values of all the atoms of a molecule referred to ***r***_origin_ = (0,0,0)
|***Q*_**2**_**|(**1**)	The magnitude of ***Q*_2_** computed at ***r*** = (0, 0, 1 Å)
***Q*_**2**_**(**1**)**_*zz*_**	The value of *Q*_20_ = *Q*_*zz*_ computed at = (0, 0, 1 Å)

a The components of the ***Q*_2_** tensor are given in atomic units (*ea*_0_^2^).

### Interpretation of Quadrupole Tensor Components as Aromaticity Descriptors

The interpretation of the quadrupole tensor components in a DMA analysis is directly related to the spatial distribution of electron density. The quadrupole tensor measures the anisotropy of charge distribution in a molecule, that is, how the electron density deviates from spherical symmetry. Since the quadrupole involves weighted integrals by *r*^2^ (e.g., eq 2), regions further from the center contribute more significantly to its values. Consequently, the electron delocalization (characteristic of aromatic systems) increases the magnitude of the quadrupole tensor components.

However, multipole moments are sensitive not only to the electron density distribution but also to the molecular symmetry. In highly symmetric systems, such as benzene in its optimized geometry, multipolar contributions from different atomic centers (e.g., the carbon and hydrogen atoms) can interfere, leading to a partial cancellation of the tensor components. This symmetry-induced cancellation may result in lower non-normalized values for the quadrupolar tensor, even in strongly aromatic molecules. Therefore, while increased electron delocalization is generally expected to correlate with higher descriptor values, caution is necessary when interpreting these values in highly symmetric systems. One strategy to address this issue is to normalize the descriptor values by using benzene as a reference. Benzene, with its high symmetry and well-established aromatic character, serves as a standard benchmark. Solà and co-workers have already discussed this normalization approach.^66^

It is important to note that the sign of a specific descriptor does not necessarily indicate “more” or “less” delocalization, but rather the orientation of the electron distribution with respect to a reference axis. In practical terms, considering the ***Q*_2_** component involved in the combination 3*z*^2^–*r*^2^ (for instance, *Q*_20_ in the spherical notation or our descriptor *Q*_*zz*_), a positive value generally indicates that the electron distribution is more concentrated along the *z*-axis, resulting in a “prolate” electron cloud. In contrast, a negative value suggests greater extension or density in the direction perpendicular to the *z*-axis (“oblate” or disc-like). In other words, the quadrupole sign conveys information about the preferential orientation of the electron density—whether it is elongated along a given axis or spread out in the perpendicular plane. However, if the spatial extent of the electron density (i.e., its delocalization) changes, what is observed is an increase in the magnitude (non-normalized value) of the components, regardless of the sign.

Since the quadrupole tensor has multiple components (for instance, *Q*_20_, *Q*_22*c*_, etc.), each provides information about anisotropy along specific directions. Nevertheless, the fundamental interpretation remains consistent: (i) the sign indicates whether the charge distribution is elongated or flattened relative to the chosen axis, and (ii) the non-normalized value of the descriptor reflects the degree of delocalization.

To sum up, increasing aromaticity–and, consequently, electron delocalization–usually manifests as an increase in the non-normalized magnitude of the DMA quadrupole tensor components *Q*_2_ if the modification is on the ring plane. However, caution is required in highly symmetric systems as the symmetry-induced cancellation of quadrupole tensor components can yield lower values than expected. A possible solution to overcome this issue is to normalize the descriptor values by using benzene as a reference, as done here. Meanwhile, the sign itself reflects the geometry of the distribution (elongated and perpendicular to the reference plane or flattened and parallel to it) rather than the degree of delocalization.

### How to Obtain the ***Q*_2_** DMA Tensor Components for Computing the New Aromatic Descriptors

The geometry of all the molecular structures investigated in this work was converged in the work of Solà and co-workers.^18,19^ They were obtained at the B3LYP/6-311G++G(d,p) level.

The molecular axis must be properly aligned before computing the descriptors, as their orientation can significantly affect the results. The *z*-axis needs to be perpendicular to the molecular plane to determine the descriptors accurately. The NOSYMM keyword must be used in the Gaussian program to ensure it does not alter the molecular orientation during the single-point calculation of the electron density.

The single-point calculations in this work using the geometries by Solà et al. employed the Mo̷ller–Plesset perturbation theory at the second-order (MP2) electronic structure method^67^ implemented in the Gaussian 09 Revision A.02 package. MP2 is an ab initio method that does not suffer from some of the well-known problems of DFT, starting from the ambiguity of choosing an appropriate exchange-correlation functional.^68^ The 6-311++G(d,p) Gaussian basis set was used, resulting in the MP2/6-311++G(d,p) level for the single-point calculations. The keyword DENSITY = MP2 in the Gaussian input file was set to produce the MP2 molecular electronic density to be used in the DMA partition of the electron density.

The DMA partition of the electron density of each molecule in this work was computed from the Gaussian-formatted checkpoint files (*.fchk) with the GDMA2 software.^51^ The computational protocol of the ***Q*_2_**-based aromatic indices (Table 1) is described in detail in the Supporting Information. A Python script is provided to generate the GDMA2 input and to compute the proposed descriptors – see the Supporting Information. Other electronic structure packages compute the DMA electric multipoles directly, thus the necessary ***Q*_2_** components, making the computation of the proposed aromaticity descriptors highly accessible and straightforward.

The computation of the DMA multipoles, thus of the components of the ***Q*_2_** second-rank tensor, is not restricted to MP2. In principle, any electronic structure method can be used. However, in GDMA2, the default electron density corresponds to the SCF density. To compute the multipoles using other types of electron density from Gaussian, it is necessary to specify the density explicitly. For instance, the command “File *file.fchk* DENSITY CC” should be used to extract the Coupled-Cluster (CC) density from a Coupled-Cluster Singles and Doubles (CCSD) calculation, instead of the default SCF density. The *file.fchk* refers to the formatted checkpoint file. In previous work, we tested the accuracy of the DMA electric multipole methods using five different types of DFT functionals, MP2, MP4, and Hartree–Fock methods (HF), on molecules bearing different bonds and structures.^69^ The MP4 level was the benchmark. Overall, we found that the DMA values of the monopole (charge), dipole, and quadrupole (***Q*_2_**) electric multipoles, especially the latter, are pretty insensitive to the electronic structure level, including the type of exchange-correlation functional. Even the HF overall produced multipoles of similar accuracy, which is unsurprising, considering that the electron density is not very sensitive to the electronic correlation.^70,71^ The dependence of the DMA multipoles on the size of the basis set is minor, in most cases not exceeding 5%.

## Results and Discussion

As previously stated, some component(s) or combinations of the ***Q*_2_** DMA second-rank tensor measure electron delocalization in molecular structures, which can be associated with aromaticity. An increase in ***Q*_2_**- related values for a given molecule, when compared to benzene, for instance, does not necessarily imply that the molecule has become “more aromatic”. The idea behind the proposed ***Q*_2_**-based aromatic indices is to use them to detect electron density variations, which may or may not lead to an increase in electron delocalization in each case. We focus then on variations of the *Q*_**2**_**-**based descriptors (defined in Table 1) values in relation to a reference molecule, benzene. The reported normalized descriptor values are the computed values divided by the corresponding benzene ones. Therefore, the normalized values of the descriptors are used for examining the aromaticity trends in each test.

To evaluate the viability of the proposed six new aromatic descriptors, we use the Girona benchmark proposed by the Solà group, which comprises 14 sets of typical aromatic and nonaromatic systems.^18,19^ Tests T13 and T14 were omitted because they involve chemical reactions. The tests are depicted in Table 2. The computed numerical values of the ***Q*_2_** aromaticity descriptors in each case are presented in Tables 1S–12S of the Supporting Information.

**Table 2:**
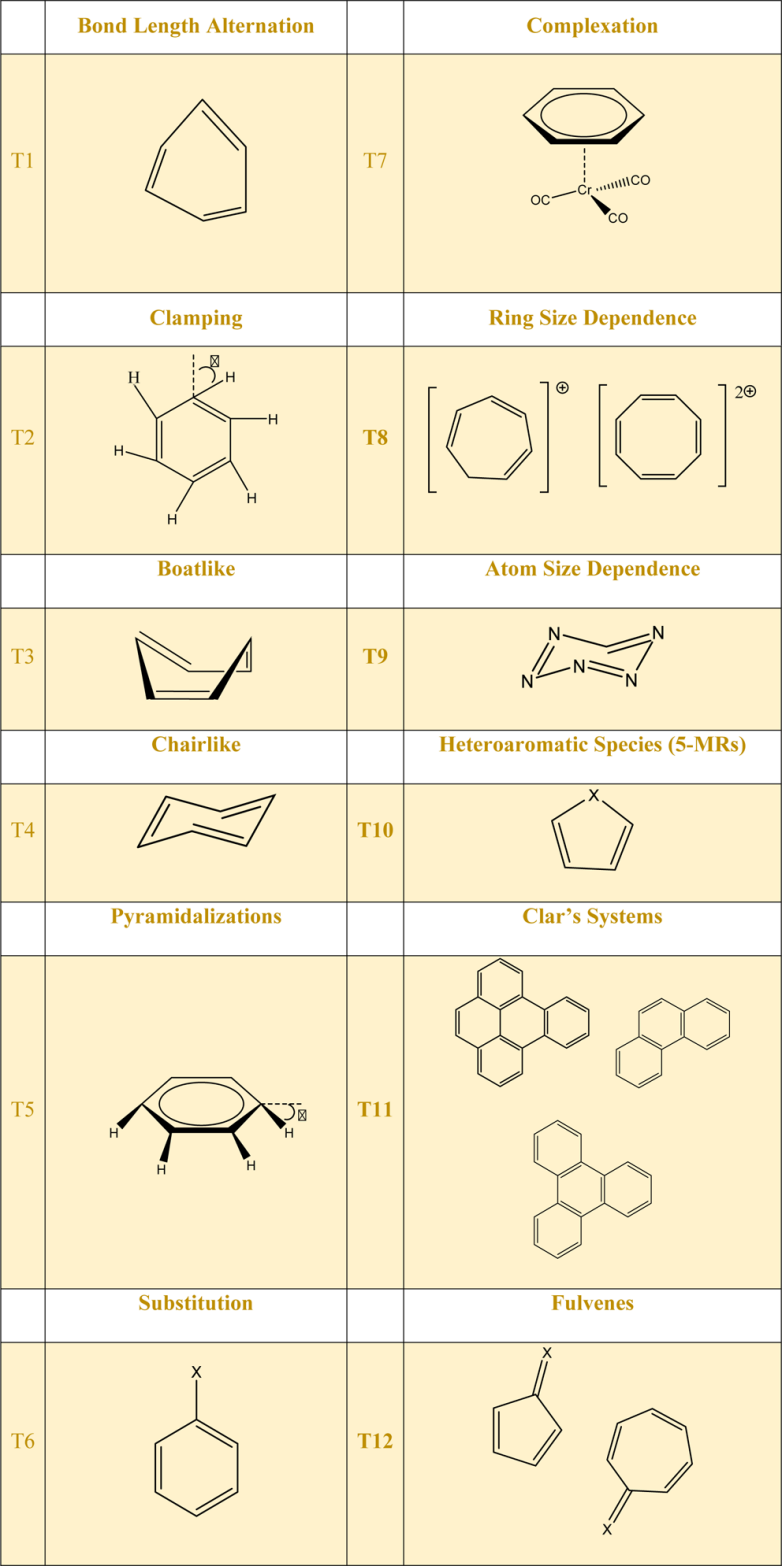
12 Aromatic Tests of the Girona Benchmark^19^^a^

a Adapted with permission from *Chem. Soc. Rev.***2015**, *44*, 6434. Copyright 2015 by the Royal Society of Chemistry.

The first test, T1, is one of the first five tests that uses benzene distortions. The reference results in each test are indicated by the red rows in Tables 1S–12S. T1 consists of modifying the bond lengths of the benzene ring by shortening double bonds and lengthening the single bonds, as shown in Table 2. In benzene, all the bond lengths between the carbon atoms in the ring are equal to 1.39 Å, resulting in a bond length difference (Δ*R*) between the two types of bonds in reference benzene equal to zero. The purpose of T1 was to investigate the influence of bond length difference on the aromaticity of this structure. As the Δ*R* value increases, the molecular structure deviates from the ideal configuration, which is expected to decrease the electron delocalization and, thus, the aromaticity.

**Figure 4:**
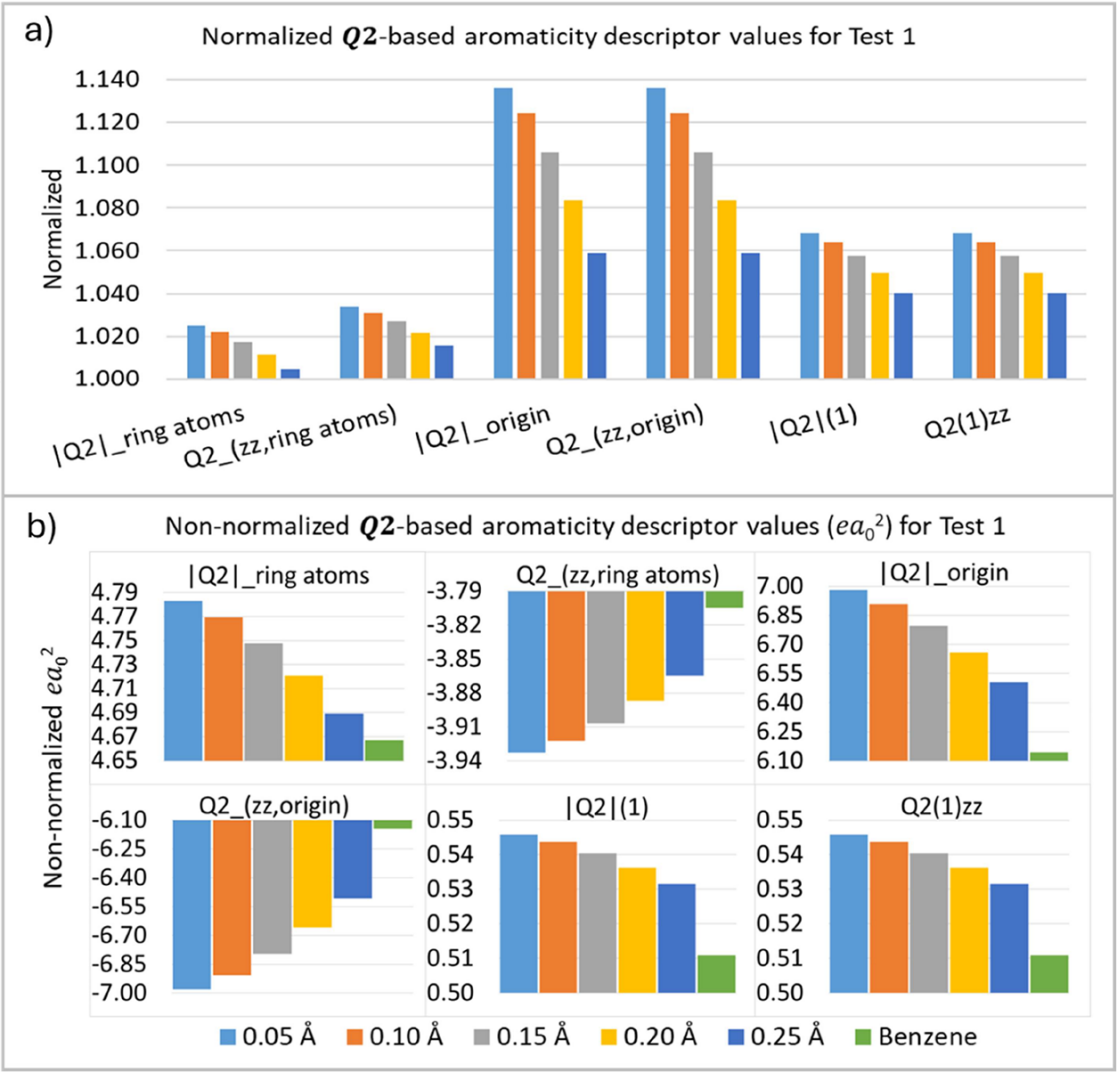
***Q*_2_**-based aromaticity indices (*ea*_0_^2^) for test 1 (T1), (a) normalized and (b) non-normalized values. The numbers at the bottom of the figure are the bond length difference (Δ*R*) values in Ångstroms.

The analysis of the Δ*R* values (Figure 4) shows that the ***Q*_2_**-based aromaticity descriptor values varied for all five distorted benzene structures, deviating from the reference value (benzene). The normalized values (Figure 4a) highlight relative deviations across different structural distortions. These results indicate that the proposed descriptors are sensitive to changes in bond lengths within the molecular structure. Moreover, these findings are consistent with the prediction that an Δ*R* increase decreases the aromaticity. However, the observed deviations in the ***Q*_2_** descriptor values were not significant, corroborating the conclusions of Soncini et al.,^42,43^ who suggested that variations of the bond lengths in benzene do not cause significant changes in the molecule’s aromaticity. These results reinforce the robustness of benzene’s aromaticity in the face of small structural perturbations and the fact that the proposed aromatic descriptors work well in this case.

**Figure 5:**
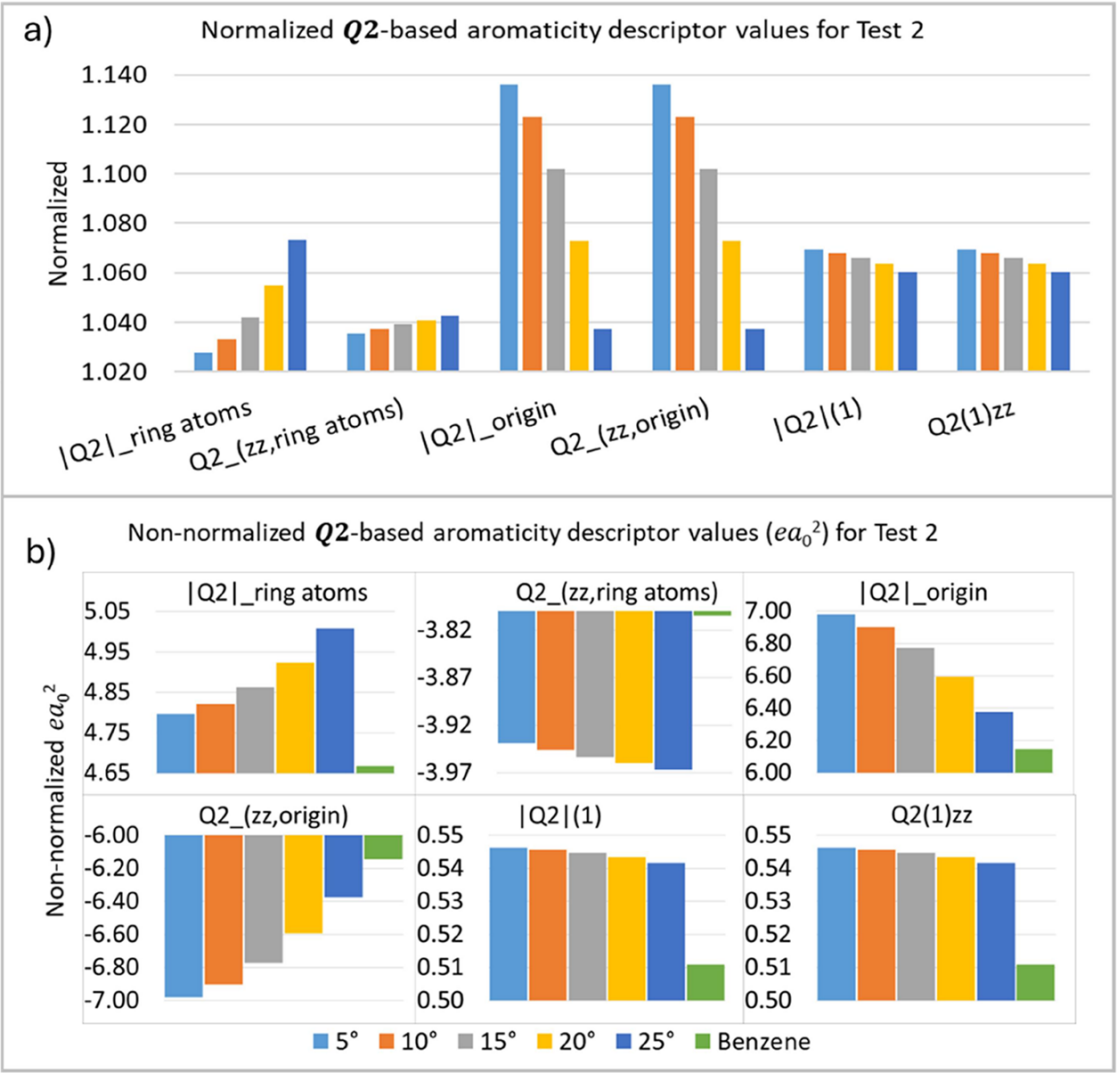
***Q*_2_**-based aromaticity indices (*ea*_0_^2^) test 2 (T2), normalized (a) and non-normalized (b) values. The values at the bottom of the plot of the α angles are in degrees.

The second test (T2) involves varying the C–H bond angle in the plane of the molecule (Table 2) to assess this variation’s impact on the system’s aromaticity. As shown in Figure 5, the ***Q*_2_** descriptors displayed significant variations in response to changes in the C–H bond angle. These deviations from the reference value indicate a pronounced loss of aromaticity in the molecular structure as they move away from the values for benzene. This variation in the ***Q*_2_** indices is due to an increase in electron density associated with the approximation of the hydrogen atoms toward the aromatic ring in the test T2, which enhances the electronic interaction within the system and, hence, modifies the descriptor values. Almost all descriptors showed a decrease in their values, indicating a reduction in aromaticity as the angle increases, in agreement with theoretical predictions. The only exceptions were the descriptors associated with the carbon atoms in the ring (*Q*_2ring atoms_ and *Q*_2*zz*,ring atoms_), although their increase was consistent with the increase in the angle. This behavior is plausible, as an increase in the angle leads to closer proximity of the sigma bonds in these atoms, resulting in more significant sigma contamination, which affects the accuracy of the aromaticity descriptors. The results (Figure 5) suggest that the ***Q*_2_** aromatic descriptors are sensitive to changes in the molecular geometry, particularly concerning the C–H bond. This behavior highlights the sensitivity of the proposed descriptors to distortions of the geometry, as subtle changes in the atomic arrangement can significantly impact the electronic properties of the system, which is an important feature to be captured by a successful aromatic descriptor.

**Figure 6:**
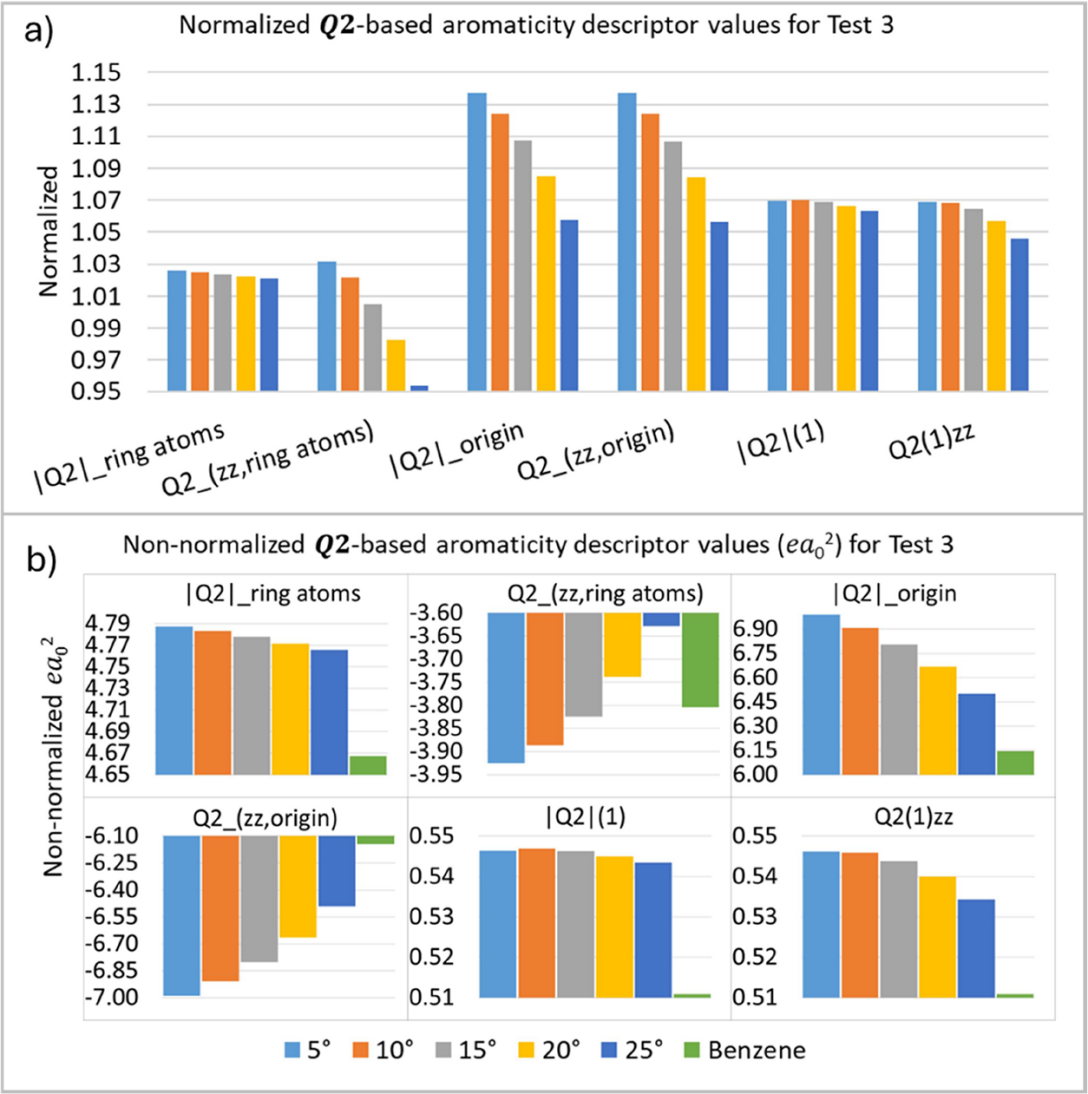
***Q*_2_**-based aromaticity indices (*ea*_0_^2^) for test 3 (T3), normalized (a) and nonnormalized (b) values. The numbers at the bottom are the variations in degrees of the C–C bond angles relative to the original plane of the benzene molecule.

In the third test (T3), the C–C bond angles relative to the original plane of the benzene molecule are varied (Table 2). The descriptors behave as expected for aromaticity, deviating from the reference benzene values, indicating a loss of aromaticity with bond variation (Figure 6).

**Figure 7:**
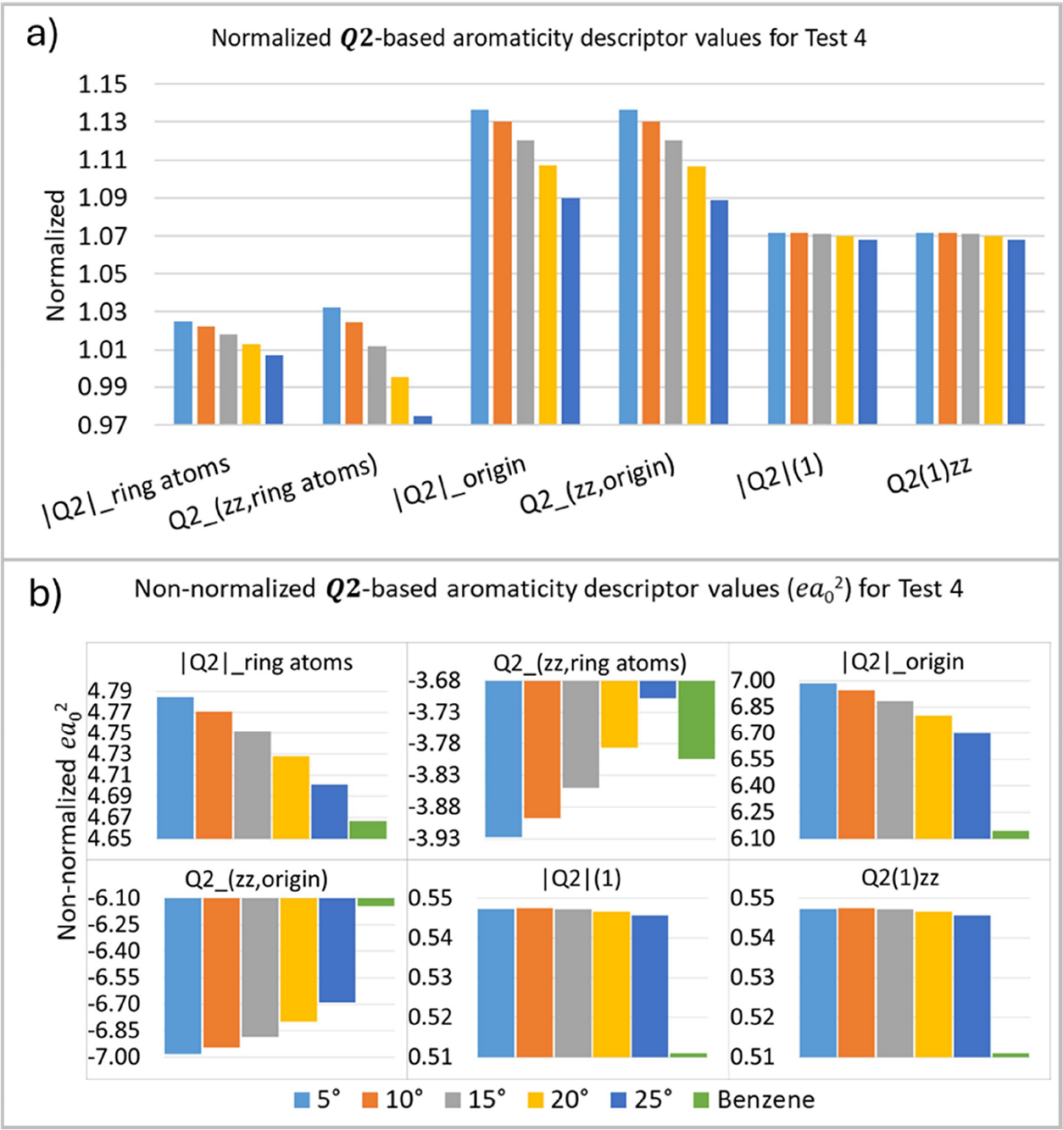
***Q*_2_**-based aromaticity indices (*ea*_0_^2^) for test 4 (T4), normalized (a) and non-normalized (b) values.

In the fourth test (T4), we investigate the variation of the C–C bond angles relative to the original plane of the benzene molecule for simulating a structure resembling a chair conformation (Table 2). The normalized plot (Figure 7a) reveals that an increase in the angle decreases the descriptor values. These results reinforce the observation that the loss of planarity affects the values of the ***Q*_2_** descriptor values. Loss of planarity is directly associated with a decrease in aromaticity,^18,19^ as shown by the changes in the descriptor values compared to the reference ones. Therefore, planarity remains critical in preserving aromaticity in molecular systems, and our aromaticity indices capture this relevant behavior.

**Figure 8:**
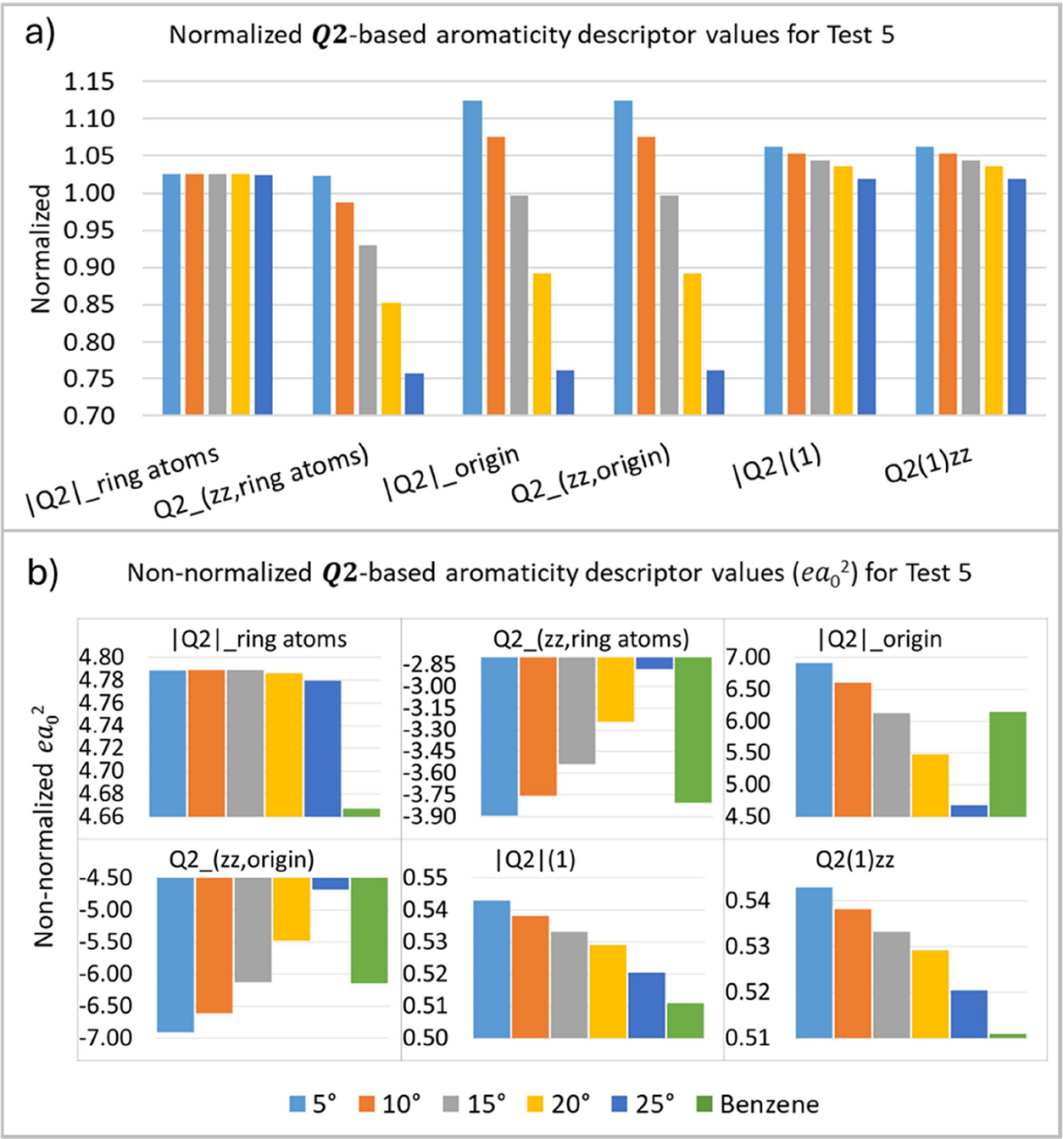
***Q*_2_**-based aromaticity indices (*ea*_0_^2^) for test 5 (T5), normalized (a) and non-normalized (b) values.

In the fifth test (T5), the out-of-plane C–H bond angle of the molecule is varied, as illustrated in Table 2, to assess the sensitivity of an aromatic descriptor to the nonplanarity of C–H bonds and their impact on the system’s aromaticity. The results showed (Figure 8) variation in the values of most of the proposed aromaticity indices, indicating that these descriptors are highly sensitive to the loss of planarity of these bonds. The normalized plot (Figure 8a) shows that an increase in the angle decreases the descriptor values.

These findings in the T1–T5 tests suggest that the ***Q*_2_** descriptors are good indicators for analyzing and quantifying molecular aromaticity, particularly when the structure’s planarity or bond lengths are disturbed. The ability of these indices to detect subtle changes in molecular geometry reinforces their utility as descriptors for evaluating the aromatic stability of nonplanar systems.

In addition to the variations in the benzene structure indicated in the first five tests performed so far, other structural modifications are introduced in the seven remaining tests of the Girona benchmark involving other chemical properties of aromatic molecules.

In the sixth test (T6), substitutions of the hydrogen atoms in the ring with electron-donating and electron-withdrawing groups are carried out, as shown in Table 2. It is known that introducing substituents in the benzene ring modifies its electron density, altering the π-delocalization and inducing partial localization of π-electrons.^19,26,72,73^

**Figure 9:**
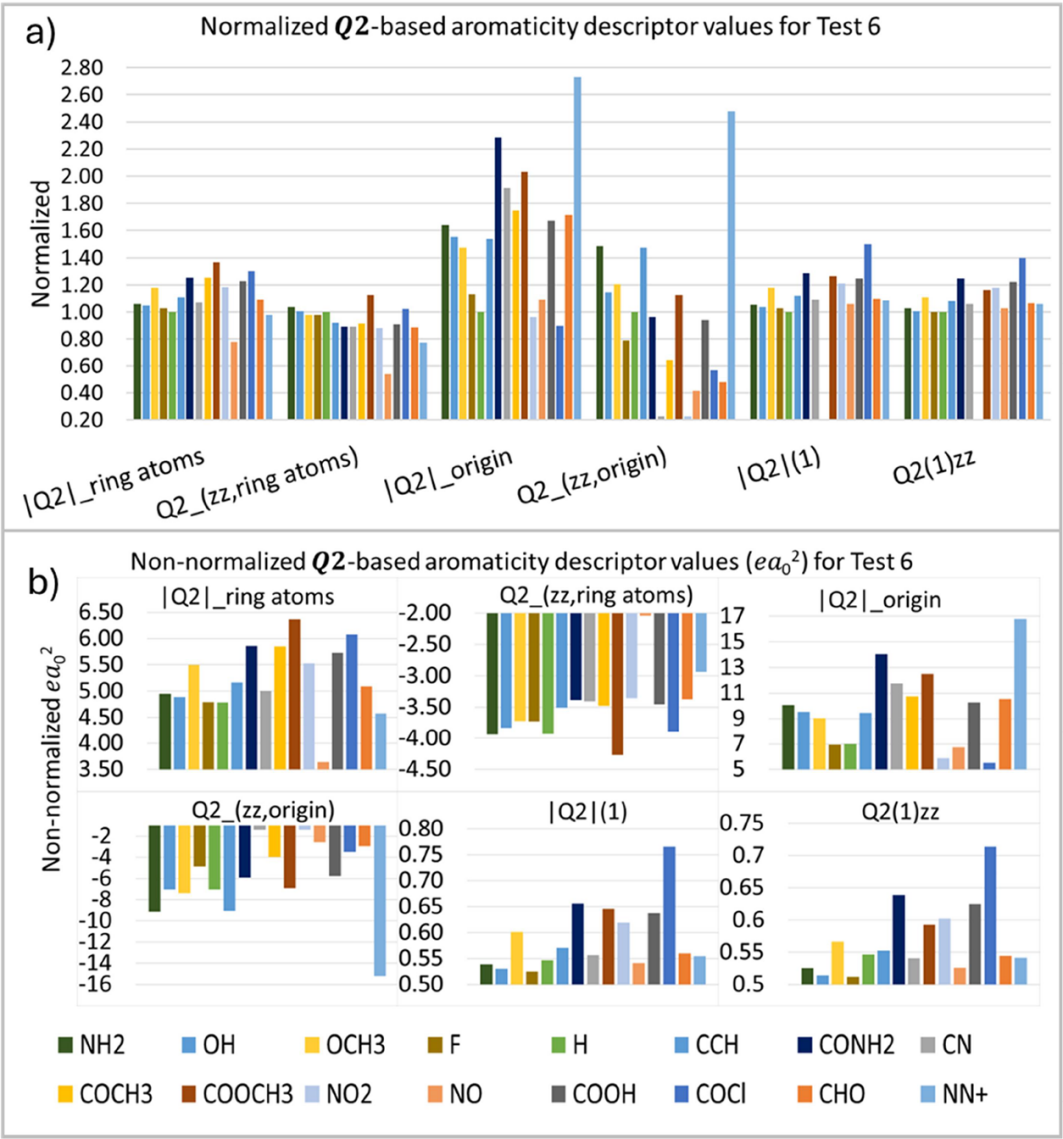
***Q*_2_**-based aromaticity indices (*ea*_0_^2^) for test 6, normalized (a) and non-normalized (b) values. “X” represents the group bonded to the aromatic ring, where X = H indicates the benzene molecule.

The analysis of the T6 test would only be complete if it considered whether these groups are electron-donating or electron-withdrawing. Other effects, such as inductive effects, mesomeric effects, and resonance interactions with the ring, would also have to be considered. Therefore, the computed ***Q*_2_** descriptor values (Figure 9) reflect each substitution’s chemical/electronic environment and behavior rather than simply indicating an expected donor/withdrawing effect. All the six ***Q*_2_**-based aromaticity descriptors in T6 show noticeable variation, indicating high sensitivity to substituent changes. Depending on the type of atoms attached to the ring, the electron density will vary according to the electronic character of each group. Electron-withdrawing and electron-donating groups modified the values of the aromaticity indices accordingly.

In Figure 9, the substituents were arranged in increasing order of electron-withdrawing effect based on the Hammett resonance constant values (σ_*R*_).^74,75^ Although the substituents do not exhibit a very clear trend, a pattern emerges, as there are inherently negative descriptors while the other descriptors tend to increase. This phenomenon occurs because electron-withdrawing substituents enhance electron density in the *xy*-plane, with the angular factor in eq 2 being negative, thus making the quadrupolar tensor more positive (e.g., Figure 2b). Therefore, while the substituents do not strictly follow the predictions of the aromaticity descriptors, they display a discernible trend.

**Figure 10:**
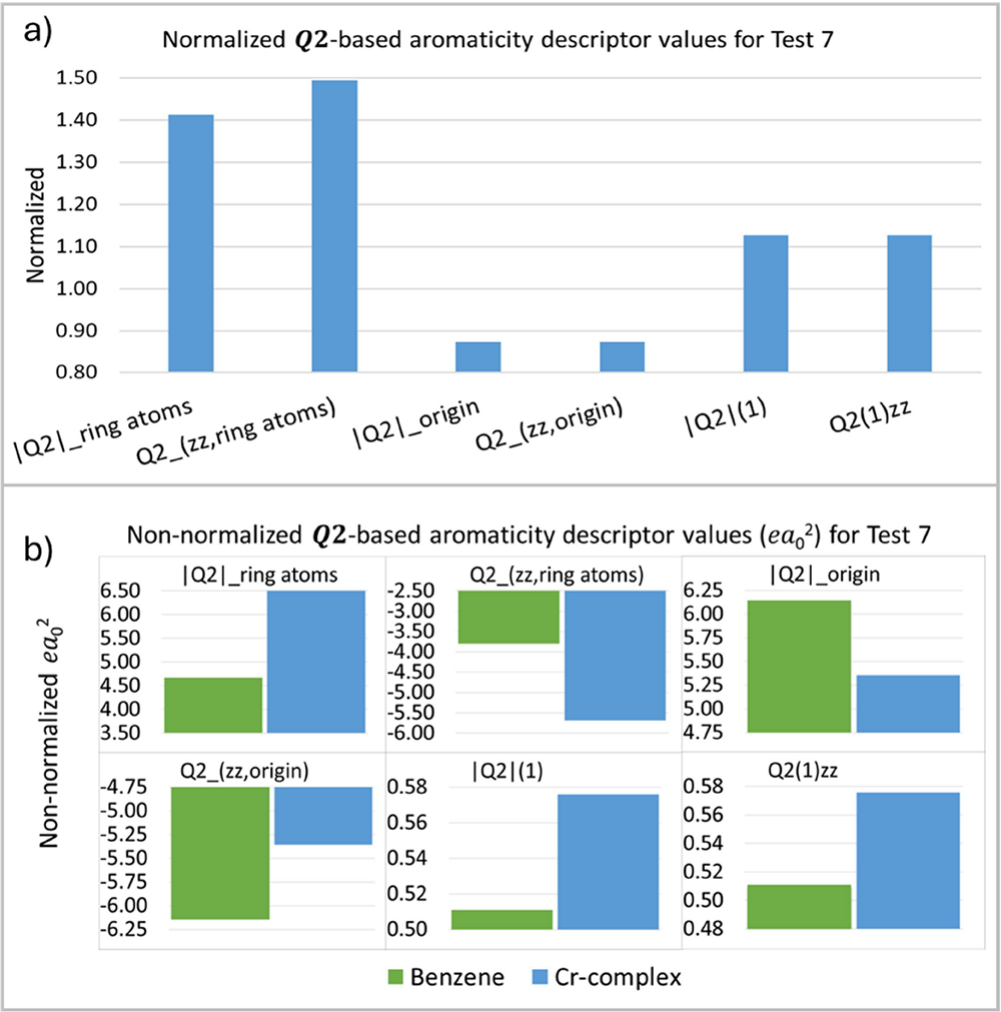
***Q*_2_**-based aromaticity indices (*ea*_0_^2^) for test 7, normalized (a) and non-normalized (b) values.

Test 7 (T7) involves the complexation of benzene with Tricarbonyl Chromium Cr(CO)_3_ (Table 2), which changes the structure of the benzene ring, thereby modifying its reactivity and aromaticity. It is patent that as the carbonyl group approaches the ring, its hydrogen atoms are directed toward it, causing structural changes. Other structural effects are also present, such as loss of planarity and variation in bond lengths. This behavior accordingly is reflected in the ***Q*_2_** aromaticity descriptor values, in which changes are readily noticeable (Figure 10), showing a surprisingly increased, but in agreement with other descriptors in the literature, higher aromaticity.^18^ However, caution is required in this test due to the different interpretations of the descriptors. Unlike the previous tests, the modification occurs perpendicular to the ring rather than being related to the *xy*-plane. Based on the benzene cone illustrated in Figure 2b, it can be observed that Cr(CO)_3_ interacts with the benzene ring through it. The CO groups, which have negative charge density, approach the cone via its edge, where the contribution to the angular factor in eq 2 is very small. In contrast, with positive charge density, the Cr atom approaches through the center of the cone, where the contribution to the angular factor in eq 2 is large and positive. As a result, the proximity of Cr(CO)_3_ tends to increase the magnitude of the aromaticity descriptors, which is consistent with other observations, except for the descriptors at the origin (see Figure 10). This finding confirms that electronic-based descriptors are the most suitable for studies of aromaticity changes upon complexation.^18,19^

**Figure 11:**
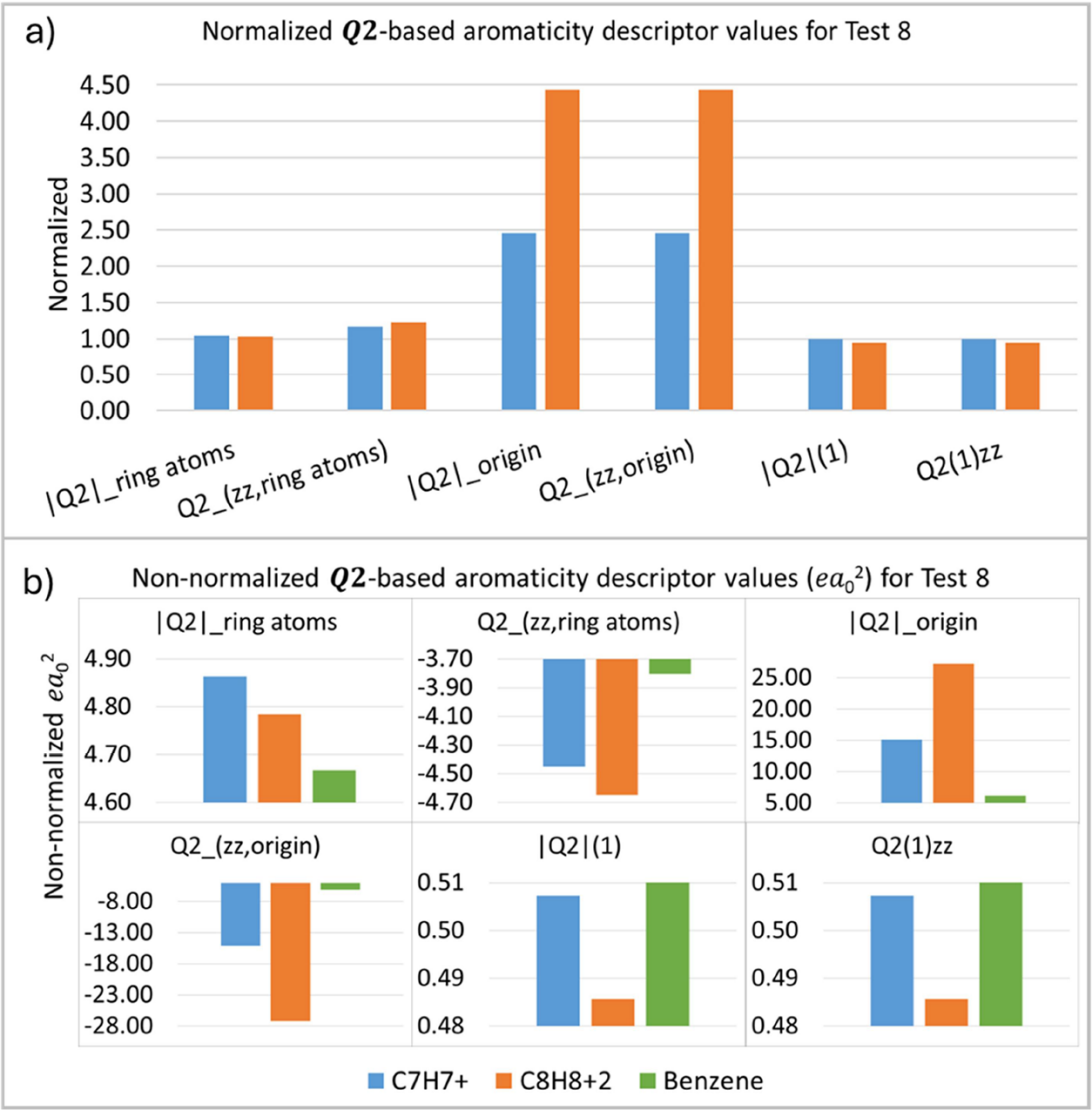
***Q*_2_**-based aromaticity indices (*ea*_0_^2^) for test 8 (T8), normalized (a) and non-normalized (b) values.

Another structural variation not previously evaluated in the first five tests is assessed in test 8 (T8). This test involves varying the size of the aromatic ring. Besides the six-membered ring of the prototypical aromatic system, benzene, which is the reference for our discussion, seven- and eight-membered rings, C_7_H_7_^+^ and C_8_H_8_^+2^, respectively, are tested here (Table 2). Aromaticity theory suggests that as the number of atoms in the ring increases, the effect of electron delocalization decreases, and consequently, the aromatic character of the structure diminishes.^18,19^ Therefore, not only a variation in the *Q*_2_ descriptor values and a gradual decrease in these values in the C_6_H_6_ > C_7_H_7_^+^ > C_8_H_8_^+2^ ordering are expected. Figure 11 indicates that the values of the descriptors follow this expected trend, with the only exceptions being |***Q*_2_**|_**zz,ring atoms**_**,***Q*_**2zz,origin**_, and |***Q*_2_**|**_origin_**. As the sigma electrons remarkably influence these descriptors, this result is expected. The descriptor |***Q*_2_**|**_zz,ring atoms_** did not follow a clear trend, likely due to the slight difference observed between benzene and C_7_H_7_^+^, which exhibited only a slight decrease of 0.009. Not only is there a patent variation in the majority of the descriptor values, but this decrease suggests that the expected sequence of “increased ring size/decreased delocalization” holds: the larger the ring, the lower the electron delocalization, and, consequently, the lower the ***Q*_2_** aromaticity descriptor values. Our results indicate this trend in the |***Q*_2_**|**_ring atoms_**, ***Q*_2_**(1), and the ***Q*_2_**(1)_*zz*_ descriptors, as shown in Figure 11. Confirming Sakai’s predictions, the decrease in these values is slight but perceptible, following the reduction of electron delocalization in this case.^76^

**Figure 12:**
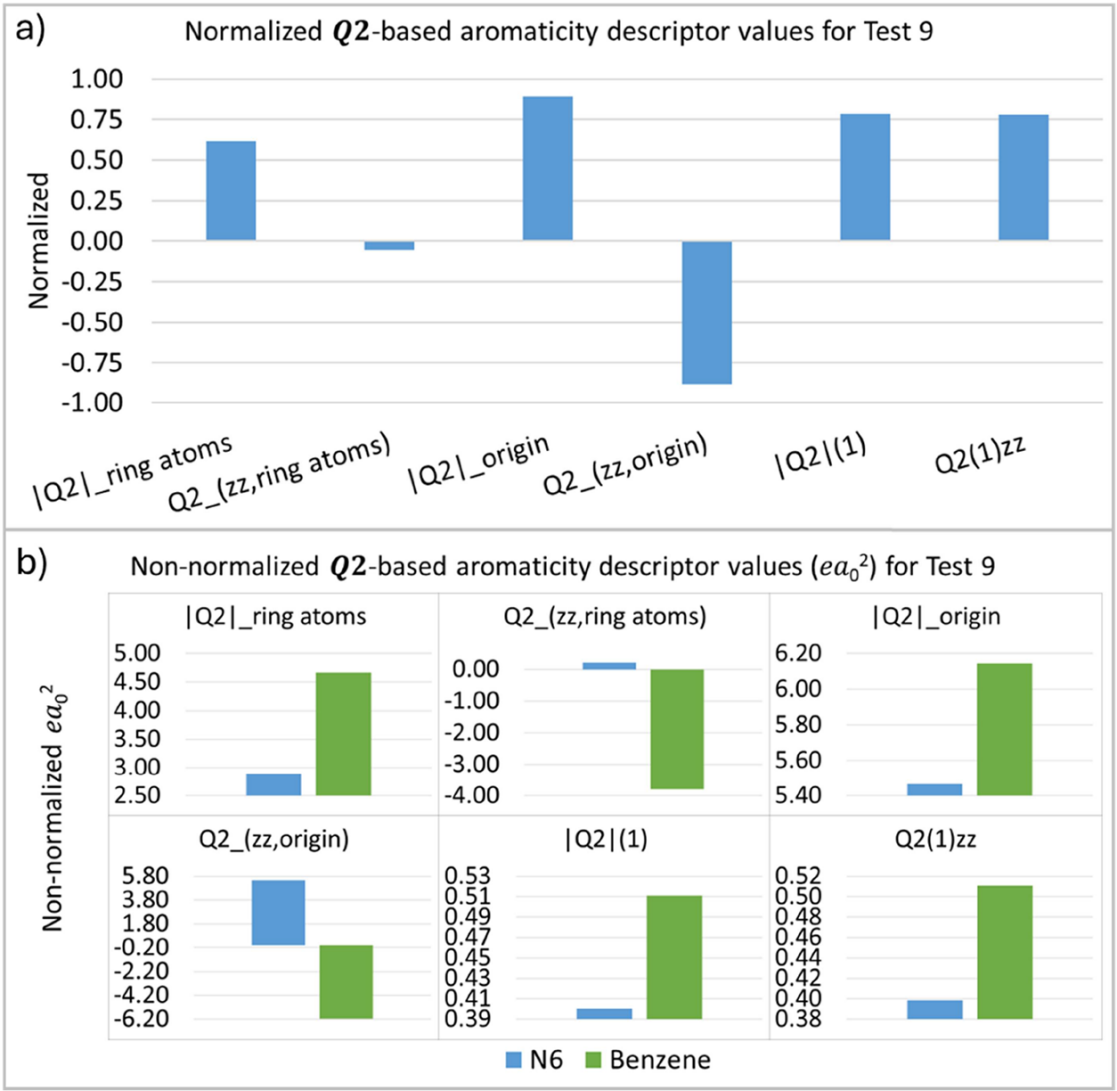
***Q*_2_**-based aromaticity indices (*ea*_0_^2^) for test 9, normalized (a) and non-normalized (b) values.

The next test (T9) also examines structural variations by replacing carbon atoms with larger atoms and examining the impact on the degree of aromaticity. The proposed structure is the N_6_ molecule (Table 2). Previous studies^76^ show that the planar N_6_ molecule is less aromatic than benzene, and Solà and collaborators anticipate a further decrease in this property for the N_6_ molecule in the “chair” configuration. Our results also reproduce this trend (Figure 12).

**Figure 13:**
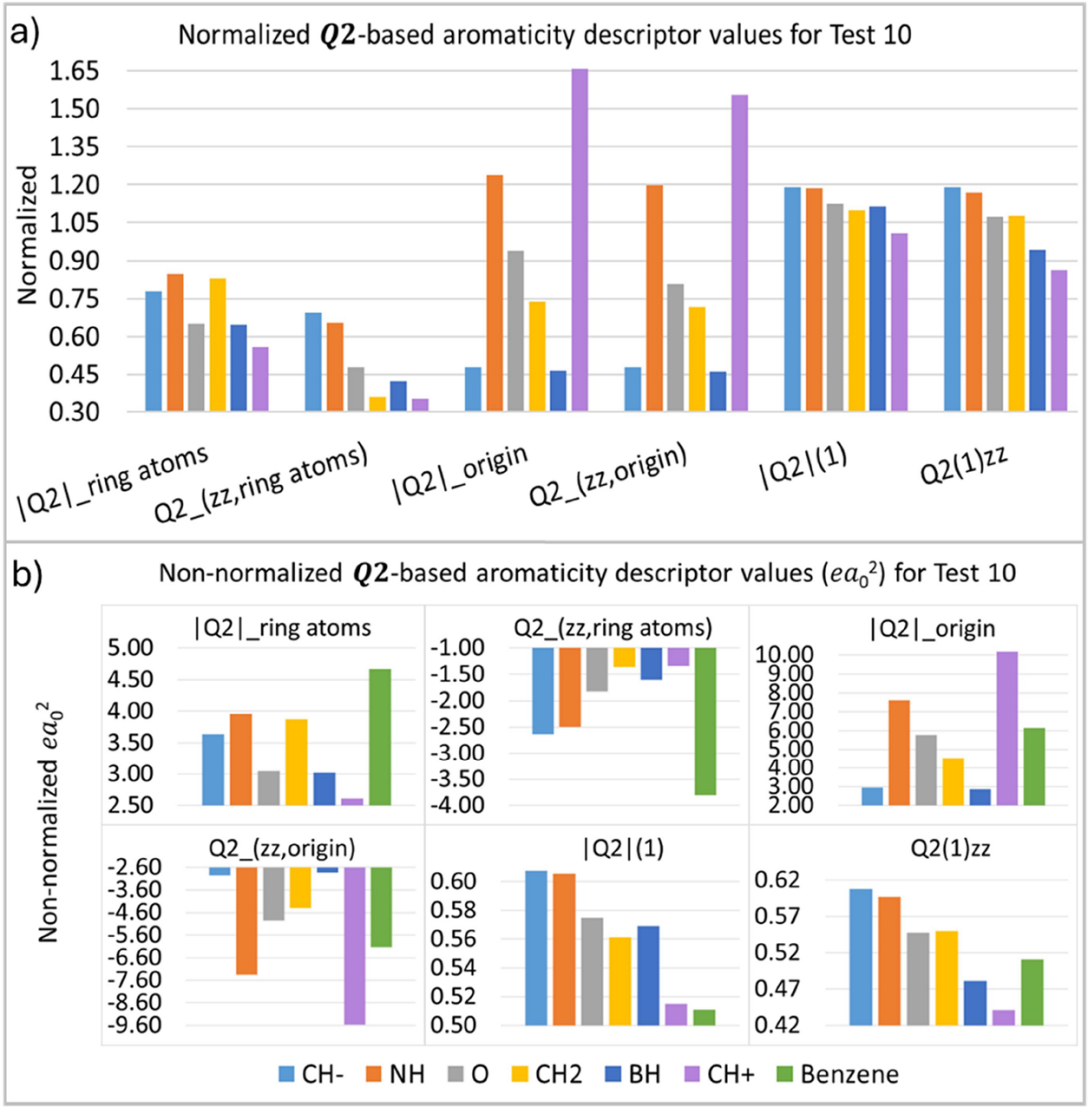
***Q*_2_**-based aromaticity indices (*ea*_0_^2^) for test 10, normalized (a) and non-normalized (b) values. “X” represents the group of heteroatoms inserted into the aromatic ring.

For test 10 (T10), heteroaromatic molecules were proposed (Table 2). As theoretical data suggest,^26^ the CH^–^ negative fragment induces a higher electron density in the ring and should, therefore, show the largest values of our aromatic descriptors. On the other hand, the CH^+^ positive fragment is expected to show the lowest values. As depicted in Figure 13, the values obtained for the tested fragments follow similarly the order proposed by Cyrański et al., namely, CH^–^ > NH > O > CH_2_ > BH > CH^+^, for almost all descriptors, except for few substituents that occupy different positions in this ordering for each descriptor. For |***Q*_2_**|**_ring atoms_** CH_2_ and O is inverted and CH^–^ is in the middle. For ***Q***_**2**(***zz***,**ring atoms**)_ and ***Q*_2_**(**1**), BH is in the middle. For |***Q*_2_**|**_origin_** and ***Q***_**2**(***zz***,**origin**)_. CH^+^ and CH^–^ and inverted, with BH the lower. For ***Q*_2_**(**1**)**_*zz*_** CH_2_ and O are inverted.

**Figure 14:**
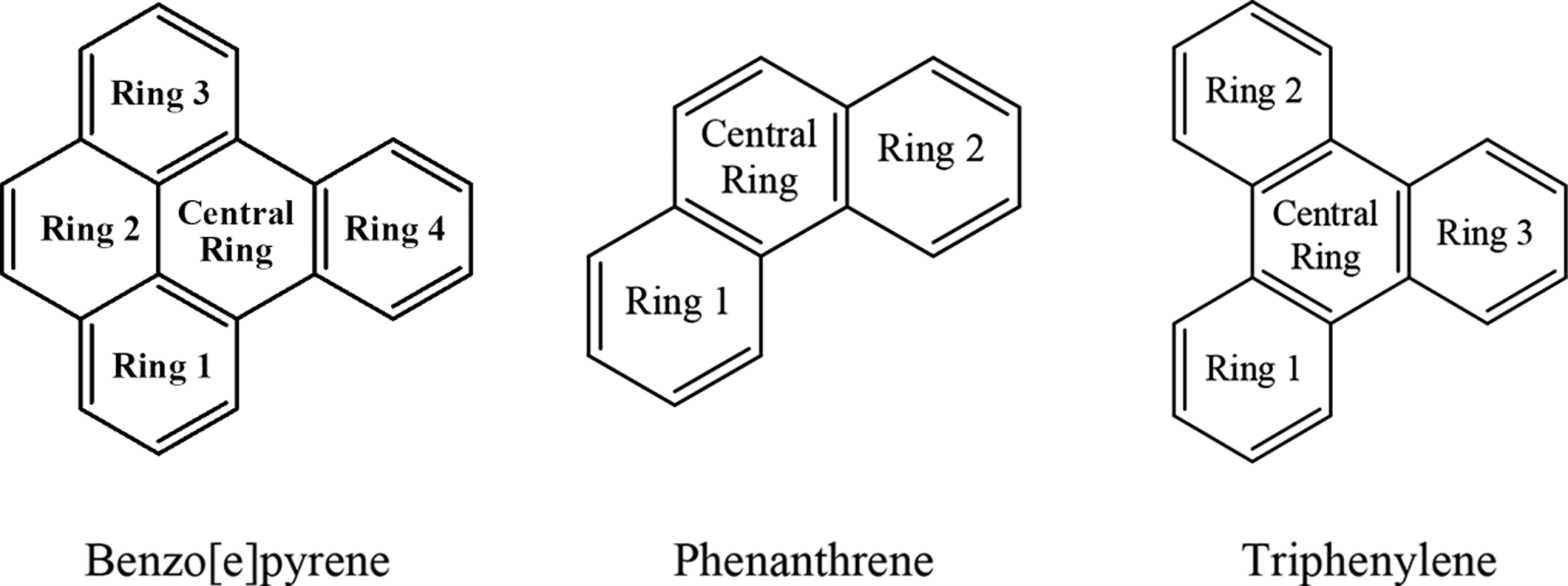
Polycyclic molecule structures for test 11 (T11).

**Figure 15:**
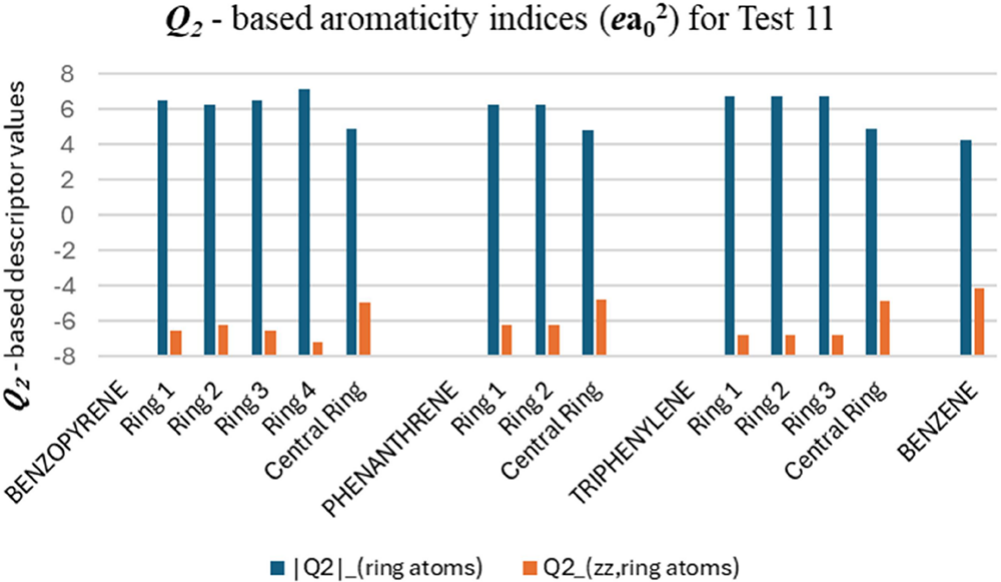
***Q*_2_**-based aromaticity indices (*ea*_0_^2^) for test 11.

Test 11 (T11) proposes an aromaticity analysis of three fused (polycyclic) aromatic ring molecules (Table 2 and Figure 14). Clar’s rule allows for predicting the distribution of aromatic π-sextets in polycyclic aromatic hydrocarbons (PAHs), indicating which rings exhibit higher or lower aromaticity within a given molecule.^77^ According to Clar’s rule, the central ring will present a smaller electronic delocalization.^78^ In the T11 test, benzene is a fully benzenoid system with one Clar’s sextet. Phenanthrene has two Clar’s sextets localized in rings 1 and 2. Therefore, outer rings (aromatic sextets) are expected to have a larger local aromaticity than the central ring with localized double bonds. The descriptors analysis confirms this prediction (Figure 15), revealing lower values for the central ring and equivalent values for rings 1 and 2, which are symmetric. Triphenylene is a fully benzenoid system with three Clar’s sextets symmetrically distributed across rings 1, 2, and 3. These rings are expected to exhibit greater local aromaticity compared to the central ring (an empty ring). This expectation is confirmed by the descriptors, where the outer rings present identical values and are higher than those of the central ring. In benzo[e]pyrene, three Clar’s sextets are localized in rings 1, 3, and 4. Ring 2, which contains a localized double bond, and the central ring (empty ring) are expected to have lower relative aromaticity. The descriptors confirm this trend, indicating that the central ring is the least aromatic, followed by ring 2. Rings 1 and 3 exhibit identical descriptor values due to symmetry, while ring 4 has the highest value due to minimal interference from less aromatic regions. Rings 1 and 3 share a boundary with rings that are not wholly aromatic (ring 2, which has a localized double bond, or the central empty ring), which may slightly attenuate their delocalization. In contrast, despite also being fused with the central empty ring, ring 4 maintains a Clar’s sextet that remains uninterrupted mainly by less aromatic regions since ring 2 is not directly connected to ring 4. Thus, our analysis indicates that rings containing aromatic π-sextets are the most aromatic, as predicted by Clar’s rule, since these π-sextet-containing rings exhibit the highest non-normalized descriptor values.

Furthermore, Clar’s rule can be employed to compare different molecules, indicating that a molecule with more isolated π-sextets tends to stabilize more by aromaticity. In a qualitative comparison among the analyzed molecules, benzene, with only one sextet, is less aromatic than phenanthrene, with two sextets. Triphenylene and benzo[e]pyrene contain three sextets; however, triphenylene, a fully benzenoid system, exhibits greater global aromaticity. Although ring 4 of benzo[e]pyrene has the highest local descriptor value, the average descriptor values of triphenylene are higher than the benzo[e]pyrene mean descriptor, indicating its greater aromaticity due to its symmetry and the larger number of uninterrupted sextets.

It is important to note that in PAHs, peripheral rings with well-defined Clar sextets show enhanced π-electron delocalization, leading to increased descriptor values. In contrast, central rings, which cannot accommodate an isolated sextet, participate only in a resonance network, preventing significant π-electron density intensification. As a result, descriptor values for these central rings remain comparable to those of benzene, which serves as a reference for an aromatic system. In summary, within a single molecule, the most stable structure is the one with the highest number of disjoint aromatic π-sextets, and central rings tend to be less aromatic than peripheral rings in a given molecule. However, when comparing different molecules, one cannot assert that the central ring is universally less aromatic. Rather, the aromaticity of rings containing Clar sextets is enhanced. Thus, although the central rings exhibit descriptor values comparable to those of benzene, they reflect in a Clar system a moderate local aromaticity without the intensification associated with isolated sextets.

**Figure 16:**
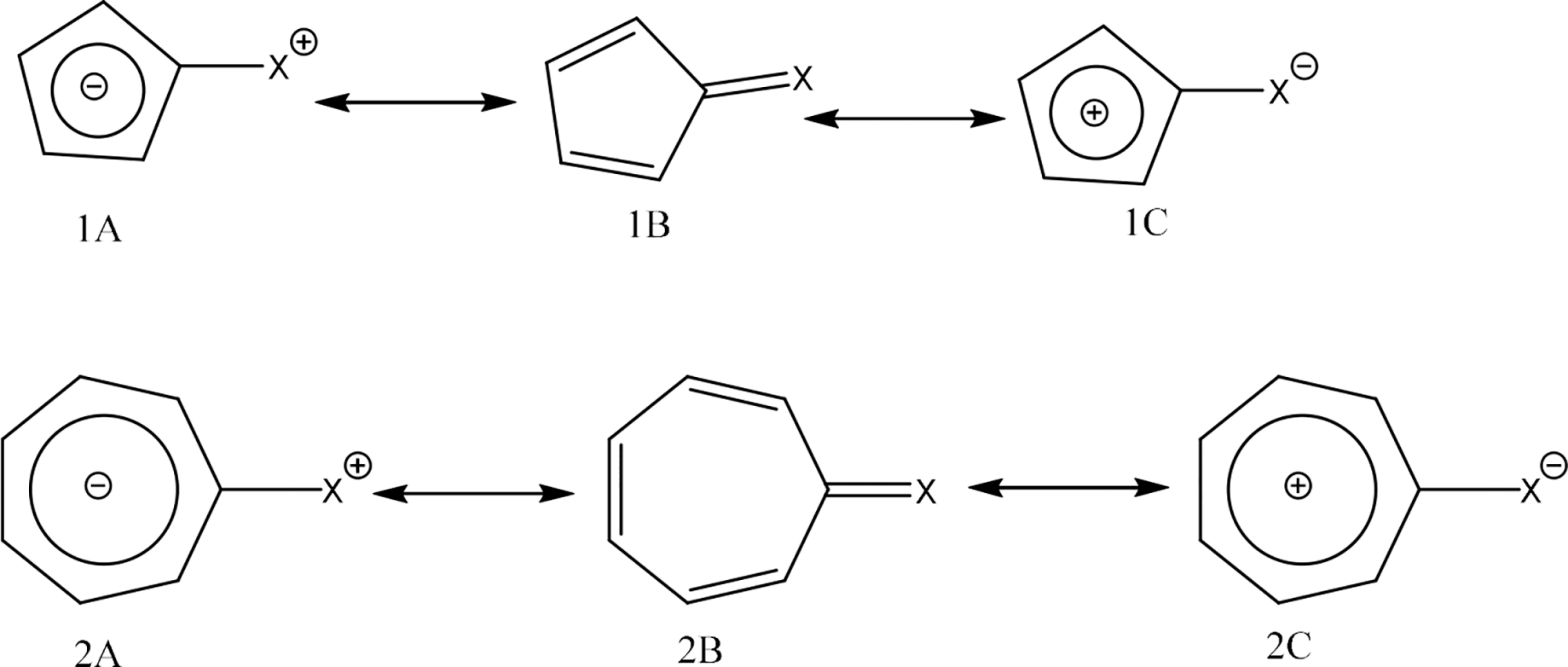
Resonance structures of penta- and hepta-fulvenes.

Finally, test 12 (T12) considers the aromaticity of penta- and hepta-fulvenes. Five different substituents, BH^2–^, O, NH, NH_2_^+^, and CH_2_, are proposed (Table 2). For this test, the analysis of the molecules depends on understanding the existing resonance structures for the five- and seven-atom rings. These structures are illustrated in Figure 16.

The predominance of each structure will depend on the characteristic of the ligand X in each case (electron-donating or withdrawing). Electron-donating substituents stabilize structures 1A and 2A, whereas electron-withdrawing groups favor the predominance of structures 1C and 2C. This relationship agrees with findings reported by various authors who utilized magnetic and structural aromaticity descriptors. These previous studies indicate that electron-donating substituents enhance the aromaticity of penta-fulvenes by increasing the contribution of the 6π-electron structure (1A). Conversely, these substituents reduce the aromatic character of the seven-membered ring (7-MR) in hepta-fulvenes by promoting the 8π-electron structure (2A).^79−82^ Electron-withdrawing substituents exhibit the opposite effect, favoring the 4π-electron structure (1C) in penta-fulvenes and the 6π-electron structure (2C) in hepta-fulvenes.

**Figure 17:**
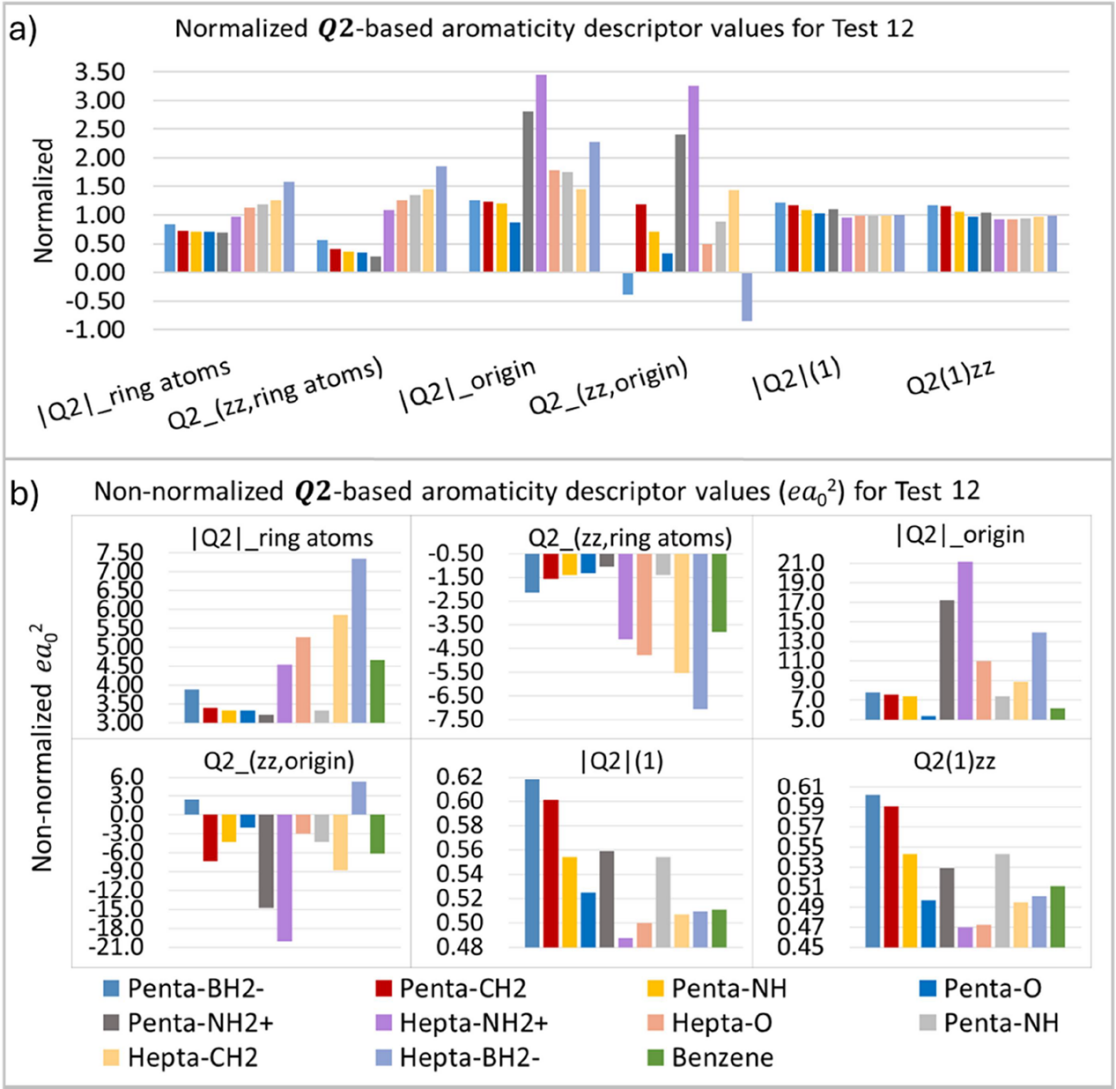
***Q*_2_**-based aromaticity indices (*ea*_0_^2^) for test 12, normalized (a) and non-normalized (b) values.

The electron-donating substituents increase the electron delocalization, thus the ring’s aromaticity. Therefore, the following aromaticity ordering is expected for the penta-fulvenes: BH^2–^ > CH_2_ > NH > O > NH_2_^+^. The opposite trend is suggested for hepta-fulvenes. According to Figure 17, it is patent that the ***Q*_2_** aromaticity descriptors partially follow the order suggested by the literature. Examining the descriptor plot in Figure 17a, a decrease in the normalized values can be observed until reaching the gray bar, which corresponds to penta-fluvene-NH_2_^+^. Beyond this point, a gradual increase in descriptor values is observed, corresponding to the hepta-fluvenes. The only exceptions to this trend are the descriptors computed at the origin, which exhibit variations for both hepta-fulvenes and penta-fulvenes with NH_2_^+^ and BH_2_^–^ substituents, but the predicted pattern for other substituents.

Overall, the results only partially reflect what was expected for test T12. In other words, Figure 17 shows that the expected sequence of increase in electronic delocalization (i.e., aromaticity) was only partially evidenced. Therefore, our descriptors in this case were less accurate than in previous tests.

When one analyzes the results from the Girona benchmark, it is found that the ***Q*_2_**-based descriptors follows a consistent pattern: negative values for **Q**_**2**_**zz,ring atoms**__, while all other descriptors display positive values. These negative values indicate that the electron density is more concentrated perpendicular to the *z*-axis, i.e., an “oblate” distribution. This is caused by the strong concentration of electrons along the σ bond.

However, the non-normalized aromatic descriptions show some deviations. In tests T1, T3, T4, and T7, benzene exhibits the lowest non-normalized descriptor values despite its high aromaticity. Due to its high symmetry, this is attributed to the partial cancellation of quadrupolar tensor components. Beyond benzene, as distortion increases, the loss of symmetry prevents such cancellation, and the descriptors decrease as expected. On test T2, benzene also shows the smallest values, and a reversed trend is observed for distortions in the *Q*_2_ring atoms__ and *Q*_2_*zz*,ring atoms__ indices caused by σ-contamination as increased angular distortion brings the hydrogen atoms closer to the carbon atoms. In T5, benzene yields intermediate descriptor values. In T6, the expected order of the descriptors fails. In T8, the descriptors calculated at the origin show an inversion due to an increased number of bonds contributing to the multipolar moment, although the descriptors *Q*_2_ring atoms__, *Q*_2_(1), and *Q*_2_(1)*_*zz*_* follow the expected order. The descriptors in T9 behave as expected. In T10, positional exchanges of one or two pairs of substituents are observed. In T12, while the expected order is confirmed for penta-fulvenes in *Q*_2_ring atoms__, the order for hepta-fulvenes is reversed, and the other descriptors do not satisfactorily follow the anticipated trend.

It is evident, therefore, that descriptors of symmetric compounds become problematic when compared to systems that do not experience the partial cancellation of tensor components. Therefore, as the choice of an aromaticity descriptor is inherently arbitrary, with benzene being widely regarded as the benchmark in aromaticity studies due to its high symmetry and well-defined electronic density distribution, as done above, we normalized all descriptors relative to benzene, which enables a more consistent comparison across different systems. The exception is test T11 (Clar’s systems), where we used the non-normalized descriptors.

After normalization, overall, the descriptor trends improved: in tests T1 and T9, all descriptors follow the expectations; in T2, the inversion in *Q*_2_ring atoms__ and *Q*_2_*zz*,ring atoms__ persists (again due to σ-contamination), while the remaining descriptors follow the expected order. In T3 and T4, all descriptors follow the expected order, except for the *Q*_2_(1) descriptor, in which the distortions at 10° and 5° appear reversed, but having 0.00098 and 0.00028 values for the respective tests. In test T5, the descriptors exhibit the expected behavior, except for the *Q*_2_ring atoms__ descriptor, which shows reversed values for the 5°, 10°, and 15° distortions, although the variations among them are less than 0.0001. In test T6, it was observed that electron-withdrawing groups tend to make all descriptors more positive, and only descriptors *Q*_2_*zz*,ring atoms__ and *Q*_2_*zz*,origin__ fail. In the test T7, the change does not occur in the plane, and the interpretation is different: the proximity of Cr(CO)_3_ is expected to increase the descriptors, which does not happen only for the descriptors computed at the origin. In test T8, a reversal of the order was observed for the origin descriptors, attributable to the enhanced quadrupolar contribution at the center of the ring due to the increased number of atoms involved. In contrast, the remaining descriptors followed the expected order. In test T10, the positional switching behavior observed in the ordering with one or two pairs of substituents is maintained. For test 11, benzo[e]pyrene and triphenylene are reversed for the origin descriptors, with the expected order for the ring atoms descriptors and unsatisfactory for 1Å descriptors. Finally, in test T12, descriptors follow the expected order, except at the origin, where variations occur for the NH_2_^+^ and BH_2_^–^ substituents in hepta- and penta-fulvenes.

In Table 3, we evaluate the six ***Q*_2_**-based descriptors according to the 12 aromaticity tests. The table is inspired by a similar one suggested by the Girona group to systematize the results.^19^ A ‘green checkmark’ indicates that the descriptor follows the expected trend: the increase or decrease in a normalized descriptor value corresponds to the increase or decrease of aromaticity. A ‘red cross’ implies a significant discrepancy from the expected trend. The ‘yellow circle’ represents a minor deviation, where the descriptor does not accurately reflect the expected order of growth or decline in aromaticity.

**Table 3:**
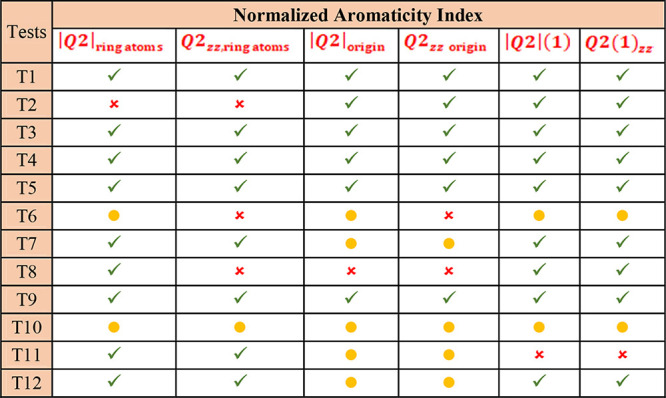
Summary of the Results From Testing the Six Proposed ***Q*_2_**-Based Aromaticity Descriptors Using the Girona Benchmark^18,19^^a^

a All descriptors have been normalized relative to benzene values. A green checkmark (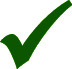) indicates that the descriptor followed the expected aromaticity trend, and a red cross (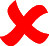) indicates a significant discrepancy. A yellow circle (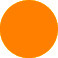) represents a minor deviation.

We obtained 65% green checkmarks, 22% yellow circles, and 13% red crosses. Considering that green checkmarks and yellow circles are favorable results (i.e., the descriptor values follow the expected trend), the results are encouraging: the proposed six descriptors passed 87% of the 12 different tests. Therefore, the proposed indices have the potential to be useful for aromaticity indicators to investigate other aromatic molecules.

## Conclusion

Cyclic electron delocalization is a key property of aromatic compounds. In this work, we proposed six new aromaticity descriptors based on the partition of the molecular electron density using Stone’s DMA method. The aromatic descriptors are based on the components of the DMA electric quadrupole ***Q*_2_** tensor, the first in the DMA multipolar expansion to have contributions from the out-of-plane electron density. Therefore, the out-of-plane components of the ***Q*_2_** tensor are particularly suited for capturing the effects of delocalized electrons. Considering the popularity of Stone’s DMA method, which is implemented in different electronic structure packages, and the possibility of using the Gaussian program with the GDMA2 software to compute the required ***Q*_2_** tensor components, the proposed aromatic indices can be easily obtained and used. In the Supporting Information, we show how to calculate them.

To assess the performance of the six ***Q*_2_**-based aromaticity descriptors, we employed 12 tests of the Girona benchmark.^18,19^ The carefully designed aromaticity tests included different distortions of the benzene framework, substitutions, complexation, ring size dependence, atomic dependence, heteroatomic species, Clar’s systems, and fulvenes. The tests thus included aromatic and nonaromatic molecular systems. The benzene molecule was used as a reference for the new aromaticity indices. The results collected in Table 3 showed that in 65% of the tests, the ***Q*_2_** descriptors followed the expected aromaticity trends. For 22% of the cases, the descriptors correctly described most of the trends, and in 13% they failed utterly. As green checkmarks and yellow circles indicate favorable results, the results are encouraging: the proposed six descriptors passed 87% of the 12 tests.

Future planned works include applying the proposed new descriptors to capture the magnetic aspects of aromaticity in PAHs, similar to the analysis performed by Leyva-Parra et al.^83^ The *Q*_**2**_**-**based aromaticity descriptor values could be correlated with magnetically induced current densities to evaluate both local and global aromatic contributions.

The proposed aromaticity descriptors are based on a chemically intuitive partition of the electron density employing the DMA electric multipolar expansion, which allows capturing the electronic delocalized effects typical of most aromatic systems. Therefore, by themselves or combined with other aromaticity descriptors, the proposed ***Q*_2_**-based descriptors can be used to rationalize and quantify various aromatic phenomena. We invite researchers from different aspects of aromaticity to test and apply the proposed descriptors.

## Supporting Information Available

The Supporting Information contains tables with numerical values of the six aromaticity descriptors for all the 12 tests (Tables 1S–12S). Figure 1S with scatter plots showing |*Q*_2_|(*x*) and *Q*_2_(*x*)*_*zz*_* versus distance *x*. It also presents the computational protocol to compute the ***Q*_2_**-based aromaticity descriptors, including illustrative input and output files of the GDMA2 program. Discussion of the provided Python codes is provided (PDF)

## Notes

All data and Python codes for this work are available on GitHub and Zenodo, and they can be accessed at 10.5281/zenodo.15020319.
