# Supplementary material for: A New Set of Aromaticity Descriptors Based on the Electron Density Employing the Distributed Multipole Analysis (DMA)
Source: ACS Omega. 2025 Apr 3;10(14):14157–75. doi: 10.1021/acsomega.4c11451 (PMC12004192; doi:10.1021/acsomega.4c11451)
Supplement: Supplementary file 1 — ao4c11451_si_001.pdf [file ao4c11451_si_001.pdf]

SUPLEMENTARY MATERIAL

A new set of aromaticity descriptors based on the electron density employing the distributed multipole analysis (DMA)

Matheus Máximo-Canadas,[1] Roberta Siqueira Soldaini Oliveira,[1] Marco Aurélio Souza Oliveira,[1] Itamar Borges, Jr[1,2,]*

[1]Departamento de Química, Instituto Militar de Engenharia (IME), Praça General Tibúrcio, 80, Rio de Janeiro, RJ, 22290-270, Brazil

[2]Departamento de Engenharia de Defesa, Instituto Militar de Engenharia (IME), Praça General Tibúrcio, 80, Rio de Janeiro, RJ, 22290-270, Brazil

* Corresponding author: itamar@ime.eb.br

SUMMARY

	PAGE
**Figure S1.** Scatter plot for $\|Q_2\|(x)$ (a) and $Q_2(x)_{zz}$ (b) values, where x is the distance from the center of the ring to the point (0,0,x), in Å. The 1 Å maximizes the descriptor value.	3S
**Table 1S.** Computed $\mathbf{Q_2}$-based aromaticity indices ($\mathbf{ea_0^2}$) for Test 1 (T1).	4S
**Table 2S.** Computed $\mathbf{Q_2}$-based aromaticity indices ($\mathbf{ea_0^2}$) values for Test 2 (T2).	4S
**Table 3S.** Computed $\mathbf{Q_2}$-based aromaticity descriptor values ($\mathbf{ea_0^2}$) for Test 3 (T3).	5S
**Table 4S.** Computed $\mathbf{Q_2}$-based aromaticity indices ($\mathbf{ea_0^2}$) for Test 4 (T4).	5S
**Table 5S.** Computed $\mathbf{Q_2}$-based aromaticity descriptor values ($\mathbf{ea_0^2}$) for Test 5 (T5).	6S
**Table 6S**. Computed $\mathbf{Q_2}$-based aromaticity descriptor values ($\mathbf{ea_0^2}$) for Test 6 (T6), "X" represents the group bonded to the aromatic ring, where X = H indicates the benzene molecule.	7S
**Table 7S.** $\mathbf{Q_2}$-based aromaticity descriptor values ($\mathbf{ea_0^2}$) for Test 7 (T7).	8S
**Table 8S.** $\mathbf{Q_2}$-based aromaticity descriptor values ($\mathbf{ea_0^2}$) for Test 8 (T8).	8S
**Table 9S.** $\mathbf{Q_2}$-based aromaticity descriptor values ($\mathbf{ea_0^2}$) for Test 9 (T9).	8S
**Table 10S.** $\mathbf{Q_2}$-based aromaticity descriptor values ($\mathbf{ea_0^2}$) for Test 10 (T10), "X" represents the group of heteroatoms inserted into the aromatic ring.	9S
**Table 11S.** $\mathbf{Q_2}$-based aromaticity descriptor values ($\mathbf{ea_0^2}$) for Test 11 (T11).	9S
**Table 12S.** $\mathbf{Q_2}$-based aromaticity descriptor values ($\mathbf{ea_0^2}$) for Test 12 (T12).	10S
**The computation protocol for obtaining the $\mathbf{Q_2}$ – based aromaticity descriptors**	11S
**Figure 2S.** A typical Gaussian input file for obtaining the benzene MP2 molecular electron density used as input of the GDMA program for computing the components of the second-rank tensor $\mathbf{Q_2}$ for obtaining the new aromatic descriptors indicated in Table 1 of the main manuscript.	11S
**Figure 3S.** An input file for the GDMA2 to compute the proposed $\mathbf{Q_2}$-based aromaticity descriptors.	12S
**Figure 4S.** An output file for the GDMA2 to compute the proposed $\mathbf{Q_2}$-based aromaticity descriptors. The colored numbers are the tensor components of $\mathbf{Q_2}$ for computing the proposed aromatic descriptors.	16S
**The GDMA2 input automatization**	17S
**The script for descriptor generation**	18S
**References**	27S

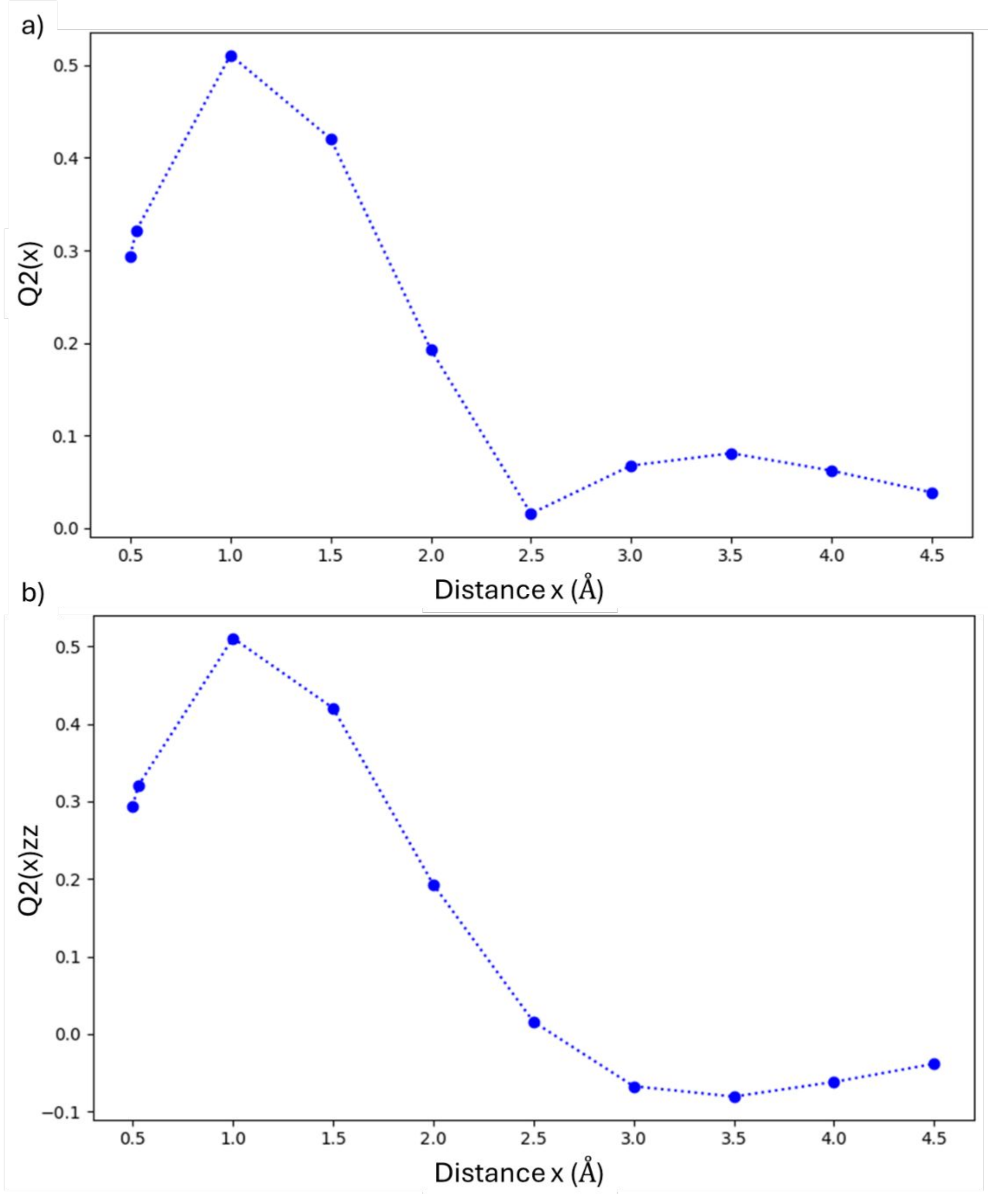

Figure S1. Scatter plot for $|Q_2|(x)$ (a) and $|Q_2|(x)_{zz}$ (b) values, where x is the distance from the center of the ring to the point (0,0,x), in Å. The 1 Å maximizes the descriptor value.

Table 1S. Computed Q_2-based aromaticity indices (ea_0^2) for Test 1 (T1)

Normalized						
***ΔR* (Å)**	$\|Q2\|_{\text{ring atoms}}$	$Q2_{\text{zz,ring atoms}}$	$\|Q2\|_{\text{origin}}$	$Q2_{\text{zz,origin}}$	$\|Q2\|(1)$	$Q2(1)_{\text{zz}}$
**0.05**	1.025	1.034	1.136	1.136	1.068	1.068
**0.10**	1.022	1.031	1.124	1.124	1.064	1.064
**0.15**	1.017	1.027	1.106	1.106	1.058	1.058
**0.20**	1.011	1.022	1.083	1.083	1.050	1.050
**0.25**	1.005	1.016	1.059	1.059	1.040	1.040
**Non-normalized**						
***ΔR* (Å)**	$\|Q2\|_{\text{ring atoms}}$	$Q2_{\text{zz,ring atoms}}$	$\|Q2\|_{\text{origin}}$	$Q2_{\text{zz,origin}}$	$\|Q2\|(1)$	$Q2(1)_{\text{zz}}$
**0.05**	4.783	-3.933	6.980	-6.980	0.546	0.546
**0.10**	4.769	-3.922	6.907	-6.907	0.544	0.544
**0.15**	4.748	-3.907	6.796	-6.796	0.540	0.540
**0.20**	4.721	-3.887	6.658	-6.658	0.536	0.536
**0.25**	4.689	-3.865	6.506	-6.506	0.532	0.532
**Benzene**	**4.667**	**-3.804**	**6.145**	**-6.145**	**0.511**	**0.511**

Table 2S. Computed Q_2-based aromaticity indices (ea_0^2) values for Test 2 (T2)

Normalized						
**α**	$\|Q2\|_{\text{ring atoms}}$	$Q2_{\text{zz,ring atoms}}$	$\|Q2\|_{\text{origin}}$	$Q2_{\text{zz,origin}}$	$\|Q2\|(1)$	$Q2(1)_{\text{zz}}$
**5°**	1.028	1.035	1.136	1.136	1.069	1.069
**10°**	1.033	1.037	1.123	1.123	1.068	1.068
**15°**	1.042	1.039	1.102	1.102	1.066	1.066
**20°**	1.055	1.041	1.073	1.073	1.064	1.064
**25°**	1.073	1.043	1.037	1.037	1.060	1.060
**Non-normalized**						
**α**	$\|Q2\|_{\text{ring atoms}}$	$Q2_{\text{zz,ring atoms}}$	$\|Q2\|_{\text{origin}}$	$Q2_{\text{zz,origin}}$	$\|Q2\|(1)$	$Q2(1)_{\text{zz}}$
**5°**	4.796	-3.939	6.980	-6.980	0.546	0.546
**10°**	4.821	-3.946	6.902	-6.902	0.546	0.546
**15°**	4.862	-3.954	6.772	-6.772	0.545	0.545
**20°**	4.923	-3.960	6.594	-6.594	0.543	0.543
**25°**	5.008	-3.967	6.375	-6.375	0.542	0.542
**Benzene**	**4.667**	**-3.804**	**6.145**	**-6.145**	**0.511**	**0.511**

Table 3S. Computed Q_2-based aromaticity descriptor values (ea_0^2) for Test 3 (T3)

Normalized						
**α**	$\|Q2\|_{\text{ring atoms}}$	$Q2_{zz,\text{ring atoms}}$	$\|Q2\|_{\text{origin}}$	$Q2_{zz,\text{origin}}$	$\|Q2\|(1)$	$Q2(1)_{zz}$
**5°**	1.026	1.032	1.137	1.137	1.069	1.069
**10°**	1.025	1.022	1.124	1.124	1.070	1.069
**15°**	1.024	1.005	1.107	1.107	1.069	1.064
**20°**	1.022	0.983	1.085	1.084	1.067	1.057
**25°**	1.021	0.954	1.058	1.057	1.064	1.046
**Non-normalized**						
**α**	$\|Q2\|_{\text{ring atoms}}$	$Q2_{zz,\text{ring atoms}}$	$\|Q2\|_{\text{origin}}$	$Q2_{zz,\text{origin}}$	$\|Q2\|(1)$	$Q2(1)_{zz}$
**5°**	4.788	-3.925	6.989	-6.989	0.546	0.546
**10°**	4.783	-3.887	6.908	-6.908	0.547	0.546
**15°**	4.778	-3.824	6.803	-6.803	0.546	0.544
**20°**	4.771	-3.738	6.666	-6.664	0.545	0.540
**25°**	4.766	-3.629	6.499	-6.492	0.543	0.534
**Benzene**	**4.667**	**-3.804**	**6.145**	**-6.145**	**0.511**	**0.511**

Table 4S. Computed Q_2-based aromaticity indices (ea_0^2) for Test 4 (T4)

Normalized						
**α**	$\|Q2\|_{\text{ring atoms}}$	$Q2_{zz,\text{ring atoms}}$	$\|Q2\|_{\text{origin}}$	$Q2_{zz,\text{origin}}$	$\|Q2\|(1)$	$Q2(1)_{zz}$
**5°**	1.025	1.032	1.136	1.136	1.071	1.071
**10°**	1.022	1.024	1.130	1.130	1.072	1.072
**15°**	1.018	1.012	1.120	1.120	1.071	1.071
**20°**	1.013	0.995	1.107	1.106	1.070	1.070
**25°**	1.007	0.975	1.090	1.089	1.068	1.068
**Non-normalized**						
**α**	$\|Q2\|_{\text{ring atoms}}$	$Q2_{zz,\text{ring atoms}}$	$\|Q2\|_{\text{origin}}$	$Q2_{zz,\text{origin}}$	$\|Q2\|(1)$	$Q2(1)_{zz}$
**5°**	4.784	-3.928	6.984	-6.984	0.547	0.547
**10°**	4.771	-3.897	6.946	-6.946	0.547	0.547
**15°**	4.752	-3.850	6.885	-6.884	0.547	0.547
**20°**	4.728	-3.787	6.802	-6.799	0.547	0.547
**25°**	4.701	-3.708	6.698	-6.691	0.546	0.546
**Benzene**	**4.667**	**-3.804**	**6.145**	**-6.145**	**0.511**	**0.511**

Table 5S. Computed Q_2-based aromaticity descriptor values (ea_0^2) for Test 5 (T5)

Normalized						
**α**	$\|Q2\|_{\text{ring atoms}}$	$Q2_{\text{zz,ring atoms}}$	$\|Q2\|_{\text{origin}}$	$Q2_{\text{zz,origin}}$	$\|Q2\|(1)$	$Q2(1)_{zz}$
**5°**	1.026	1.023	1.124	1.124	1.063	1.063
**10°**	1.026	0.988	1.076	1.076	1.053	1.053
**15°**	1.026	0.930	0.997	0.997	1.044	1.044
**20°**	1.026	0.852	0.891	0.891	1.036	1.036
**25°**	1.024	0.757	0.761	0.761	1.019	1.019
**Non-normalized**						
**α**	$\|Q2\|_{\text{ring atoms}}$	$Q2_{\text{zz,ring atoms}}$	$\|Q2\|_{\text{origin}}$	$Q2_{\text{zz,origin}}$	$\|Q2\|(1)$	$Q2(1)_{zz}$
**5°**	4.788	-3.892	6.907	-6.907	0.543	0.543
**10°**	4.789	-3.758	6.609	-6.609	0.538	0.538
**15°**	4.789	-3.539	6.129	-6.129	0.533	0.533
**20°**	4.786	-3.243	5.476	-5.476	0.529	0.529
**25°**	4.780	-2.878	4.678	-4.678	0.520	0.520
**Benzene**	**4.667**	**-3.804**	**6.145**	**-6.145**	**0.511**	**0.511**

Table 6S. Computed Q_2-based aromaticity descriptor values (ea_0^2) for Test 6 (T6), "X" represents the group bonded to the aromatic ring, where X = H indicates the benzene molecule.

Normalized						
**X**	$\|Q2\|_{ring\ atoms}$	$Q2_{zz,ring\ atoms}$	$\|Q2\|_{origin}$	$Q2_{zz,origin}$	$\|Q2\|(1)$	$Q2(1)_{zz}$
**CCH**	1.107	0.922	1.538	1.471	1.117	1.082
**CHO**	1.091	0.887	1.715	0.480	1.097	1.065
**CN**	1.073	0.895	1.913	0.225	1.090	1.058
**$COCH_3$**	1.253	0.914	1.747	0.645	1.333	1.301
**COCl**	1.303	1.023	0.896	0.568	1.499	1.397
**$CONH_2$**	1.256	0.890	2.287	0.962	1.284	1.250
**$COOCH_3$**	1.365	1.122	2.035	1.124	1.264	1.160
**COOH**	1.227	0.907	1.672	0.939	1.247	1.223
**F**	1.026	0.980	1.128	0.791	1.027	1.001
**NH_2**	1.061	1.036	1.639	1.483	1.055	1.028
**NN^+**	0.979	0.773	2.731	2.478	1.085	1.059
**NO**	0.780	0.537	1.093	0.417	1.058	1.029
**NO_2**	1.184	0.883	0.961	0.228	1.212	1.179
**OCH_3**	1.177	0.979	1.473	1.205	1.177	1.108
**OH**	1.047	1.007	1.553	1.146	1.036	1.006
**Non-normalized**						
**X**	$\|Q2\|_{ring\ atoms}$	$Q2_{zz,ring\ atoms}$	$\|Q2\|_{origin}$	$Q2_{zz,origin}$	$\|Q2\|(1)$	$Q2(1)_{zz}$
**CCH**	5.168	-3.509	9.450	-9.037	0.571	0.553
**CHO**	5.092	-3.374	10.539	-2.947	0.560	0.544
**CN**	5.006	-3.406	11.755	-1.384	0.557	0.541
**$COCH_3$**	5.849	-3.478	10.735	-3.961	0.681	0.665
**COCl**	6.079	-3.890	5.507	-3.488	0.766	0.714
**$CONH_2$**	5.860	-3.388	14.056	-5.909	0.656	0.639
**$COOCH_3$**	6.369	-4.267	12.504	-6.908	0.646	0.593
**COOH**	5.728	-3.452	10.276	-5.772	0.637	0.625
**F**	4.790	-3.728	6.932	-4.860	0.524	0.511
**NH_2**	4.951	-3.943	10.072	-9.110	0.539	0.525
**NN^+**	4.571	-2.940	16.781	-15.226	0.554	0.541
**NO**	3.639	-2.043	6.714	-2.563	0.541	0.526
**NO_2**	5.525	-3.358	5.908	-1.404	0.619	0.602
**OCH_3**	5.494	-3.723	9.050	-7.405	0.601	0.566
**OH**	4.888	-3.833	9.542	-7.042	0.530	0.514
**H**	**4.788**	**-3.937**	**7.009**	**-7.009**	**0.547**	**0.547**

Table 7S. Q_2-based aromaticity descriptor values (ea_0^2) for Test 7 (T7).

Normalized						
Molecule	$\lvert Q2\rvert_{\text{ring atoms}}$	$Q2_{zz,\text{ring atoms}}$	$\lvert Q2\rvert_{\text{origin}}$	$Q2_{zz,\text{origin}}$	$\lvert Q2\rvert(1)$	$Q2(1)_{zz}$
Cr complex	1.412	1.495	0.872	0.872	1.127	1.127
**Non-normalized**						
Molecule	$\lvert Q2\rvert_{\text{ring atoms}}$	$Q2_{zz,\text{ring atoms}}$	$\lvert Q2\rvert_{\text{origin}}$	$Q2_{zz,\text{origin}}$	$\lvert Q2\rvert(1)$	$Q2(1)_{zz}$
Cr complex	6.590	-5.686	5.357	-5.357	0.576	0.576
**Benzene**	**4.667**	**-3.804**	**6.145**	**-6.145**	**0.511**	**0.511**

Table 8S. Q_2-based aromaticity descriptor values (ea_0^2) for Test 8 (T8).

Normalized						
Molecule	$\lvert Q2\rvert_{\text{ring atoms}}$	$Q2_{zz,\text{ring atoms}}$	$\lvert Q2\rvert_{\text{origin}}$	$Q2_{zz,\text{origin}}$	$\lvert Q2\rvert(1)$	$Q2(1)_{zz}$
$C_7H_7^+$	1.042	1.169	2.457	2.457	0.993	0.993
$C_8H_8^{+2}$	1.025	1.222	4.428	4.428	0.951	0.951
**Non-normalized**						
Molecule	$\lvert Q2\rvert_{\text{ring atoms}}$	$Q2_{zz,\text{ring atoms}}$	$\lvert Q2\rvert_{\text{origin}}$	$Q2_{zz,\text{origin}}$	$\lvert Q2\rvert(1)$	$Q2(1)_{zz}$
$C_7H_7^+$	4.863	-4.449	15.100	-15.100	0.507	0.507
$C_8H_8^{+2}$	4.784	-4.648	27.211	-27.211	0.486	0.486
**Benzene**	**4.667**	**-3.804**	**6.145**	**-6.145**	**0.511**	**0.511**

Table 9S. Q_2-based aromaticity descriptor values (ea_0^2) for Test 9 (T9).

Normalized						
	$\lvert Q2\rvert_{\text{ring atoms}}$	$Q2_{zz,\text{ring atoms}}$	$\lvert Q2\rvert_{\text{origin}}$	$Q2_{zz,\text{origin}}$	$\lvert Q2\rvert(1)$	$Q2(1)_{zz}$
N6	0.619	-0.057	0.889	-0.889	0.783	0.780
**Non-normalized**						
	$\lvert Q2\rvert_{\text{ring atoms}}$	$Q2_{zz,\text{ring atoms}}$	$\lvert Q2\rvert_{\text{origin}}$	$Q2_{zz,\text{origin}}$	$\lvert Q2\rvert(1)$	$Q2(1)_{zz}$
N6	2.889	0.217	5.465	5.464	0.400	0.398
**Benzene**	**4.667**	**-3.804**	**6.145**	**-6.145**	**0.511**	**0.511**

Table 10S. Q_2-based aromaticity descriptor values (ea_0^2) for Test 10 (T10), "X" represents the group of heteroatoms inserted into the aromatic ring.

Normalized						
X	$\|Q2\|_{ring\ atoms}$	$Q2_{zz,ring\ atoms}$	$\|Q2\|_{origin}$	$Q2_{zz,origin}$	$\|Q2\|(1)$	$Q2(1)_{zz}$
**BH**	0.648	0.421	0.466	0.460	1.114	0.942
**CH^+**	0.560	0.352	1.655	1.556	1.008	0.863
**CH^-**	0.780	0.693	0.480	0.480	1.190	1.190
**CH_2**	0.830	0.358	0.739	0.716	1.099	1.076
**NH**	0.848	0.655	1.239	1.197	1.185	1.168
**O**	0.652	0.480	0.937	0.806	1.125	1.072
**Non-normalized**						
X	$\|Q2\|_{ring\ atoms}$	$Q2_{zz,ring\ atoms}$	$\|Q2\|_{origin}$	$Q2_{zz,origin}$	$\|Q2\|(1)$	$Q2(1)_{zz}$
**BH**	3.024	-1.603	2.864	-2.827	0.569	0.481
**CH^+**	2.615	-1.337	10.170	-9.559	0.515	0.441
**CH^-**	3.638	-2.637	2.949	-2.949	0.608	0.608
**CH_2**	3.872	-1.362	4.540	-4.398	0.561	0.550
**NH**	3.957	-2.492	7.613	-7.353	0.606	0.597
**O**	3.044	-1.824	5.761	-4.956	0.575	0.548
**Benzene**	**4.667**	**-3.804**	**6.145**	**-6.145**	**0.511**	**0.511**

Table 11S. Q_2-based aromaticity descriptor values (ea_0^2) for Test 11 (T11).

**Benzo[e]pryene**	$\|Q2\|_{ring\ atoms}$	$Q2_{zz,ring\ atoms}$	$\|Q2\|_{origin}$	$Q2_{zz,origin}$	$\|Q2\|(1)$	$Q2(1)_{zz}$
**Ring 1**	6.544	-6.525	3.371	3.371	0.747	0.733
**Ring 2**	6.237	-6.222				
**Ring 3**	6.544	-6.525				
**Ring 4**	7.174	-7.147				
**Central Ring**	4.941	-4.909				
**Phenanthrene**	$\|Q2\|_{ring\ atoms}$	$Q2_{zz,ring\ atoms}$	$\|Q2\|_{origin}$	$Q2_{zz,origin}$	$\|Q2\|(1)$	$Q2(1)_{zz}$
**Ring 1**	6.281	-6.231	2.429	2.429	0.946	0.911
**Ring 2**	6.281	-6.231				
**Central Ring**	4.847	-4.774				
**Triphenylene**	$\|Q2\|_{ring\ atoms}$	$Q2_{zz,ring\ atoms}$	$\|Q2\|_{origin}$	$Q2_{zz,origin}$	$\|Q2\|(1)$	$Q2(1)_{zz}$
**Ring 1**	6.772	-6.742	3.135	3.135	0.765	0.765
**Ring 2**	6.772	-6.742				
**Ring 3**	6.772	-6.742				

**Central Ring**	4.888	-4.842				

Table 12S. Q_2-based aromaticity descriptor values (ea_0^2) for Test 12 (T12).

**Normalized**						
**Molecule**	$\|Q2\|_{\text{ring atoms}}$	$Q2_{zz,\text{ring atoms}}$	$\|Q2\|_{\text{origin}}$	$Q2_{zz,\text{origin}}$	$\|Q2\|(1)$	$Q2(1)_{zz}$
**Hepta-BH_2^-**	1.573	1.858	2.270	-0.855	0.997	0.981
**Hepta-CH_2**	1.254	1.453	1.441	1.429	0.992	0.969
**Hepta-NH**	1.185	1.349	1.755	0.886	0.985	0.945
**Hepta-NH_2^+**	0.974	1.081	3.445	3.263	0.954	0.920
**Hepta-O**	1.130	1.256	1.785	0.491	0.979	0.925
**Penta-BH_2^-**	0.834	0.563	1.267	-0.393	1.210	1.178
**Penta-CH_2**	0.729	0.410	1.226	1.195	1.176	1.156
**Penta-NH**	0.714	0.367	1.205	0.706	1.085	1.063
**Penta-NH_2^+**	0.690	0.276	2.808	2.404	1.095	1.036
**Penta-O**	0.715	0.347	0.876	0.334	1.027	0.973
**Non-normalized**						
**Molecule**	$\|Q2\|_{\text{ring atoms}}$	$Q2_{zz,\text{ring atoms}}$	$\|Q2\|_{\text{origin}}$	$Q2_{zz,\text{origin}}$	$\|Q2\|(1)$	$Q2(1)_{zz}$
**Hepta-BH_2^-**	7.344	-7.069	13.948	5.254	0.509	0.501
**Hepta-CH_2**	5.851	-5.528	8.857	-8.780	0.507	0.495
**Hepta-NH**	5.529	-5.131	10.783	-5.444	0.503	0.483
**Hepta-NH_2^+**	4.544	-4.111	21.172	-20.050	0.487	0.470
**Hepta-O**	5.275	-4.778	10.971	-3.015	0.500	0.472
**Penta-BH_2^-**	3.892	-2.143	7.784	2.415	0.618	0.602
**Penta-CH_2**	3.402	-1.559	7.534	-7.342	0.601	0.591
**Penta-NH**	3.333	-1.396	7.403	-4.340	0.554	0.543
**Penta-NH_2^+**	3.221	-1.052	17.258	-14.770	0.559	0.529
**Penta-O**	3.336	-1.319	5.383	-2.054	0.525	0.497
**Benzene**	**4.667**	**-3.804**	**6.145**	**-6.145**	**0.511**	**0.511**

The computation protocol for obtaining the Q_2 – based aromaticity descriptors

Overall, there are two possible ways to compute the necessary components of the second-rank tensor $\boldsymbol{Q_2}$ to obtain the proposed $\boldsymbol{Q_2}$-based aromaticity descriptors, one using an electronic structure package that has the DMA method built-in or directly interfaced with the GDMA2 program of Stone, the other using Gaussian's output formatted checkpoint file *.fchk as part of the input for GDMA2. The first step for obtaining the DMA electric multipoles is choosing the electronic structure method to compute the electron density. Afterward, the necessary components of the $\boldsymbol{Q_2}$ tensor to derive the aromatic descriptors must be calculated.

We identified the following electronic structure programs that allow the computational of the DMA electric multipoles either as stand-alone or interfacing with the GDMA software of Stone: GAMESS (UK),[1] GAMESS (US),[2] MOLPRO,[3] Q-CHEM,[4] PSI-4,[5] and CADPAC.[6] If the Gaussian program or any other electronic structure that computes electron densities is used, in this case, the output should be converted to a Gaussian-type formatted checkpoint file *.fchk, and the GDMA2 program of Stone should be used.[7] We now discuss the second approach, used in this work – the setting of the DMA calculation is similar in the two approaches.

A typical input for a Gaussian calculation is shown in Figure 2S.

```
%Mem=3800MB
%NProcShared=6
%chk=benzene.chk # Creation of the checkpoint file for the DMA calculation using the GDMA2
program
#P MP2/6-311++G** Density=MP2 NOSYMM  # Computation of the electron density at the MP2/6-
311++G** level
Benzene
0 1

6 0.000000 1.394663 0.000000
6 1.207814 0.697332 0.000000
6 1.207814 -0.697332 0.000000
6 0.000000 -1.394663 0.000000
6 -1.207814 -0.697332 0.000000
6 -1.207814 0.697332 0.000000
1 0.000000 2.479089 0.000000
1 2.146954 1.239544 0.000000
1 2.146954 -1.239544 0.000000
1 0.000000 -2.479089 0.000000
1 -2.146954 -1.239544 0.000000
1 -2.146954 1.239544 0.000000
```

Figure 2S. A typical Gaussian input file for obtaining the benzene MP2 molecular electron density used as input of the GDMA program for computing the components of the second-rank tensor Q_2 for obtaining the new aromatic descriptors indicated in Table 1 of the main manuscript.

After running Gaussian with an input file of the type depicted in Figure 1S, the GDMA2 program must be run. A typical input file for computing the required tensor components of $\boldsymbol{Q_2}$ is shown in Figure 3S. Figure 4S depicts the corresponding output file. After collecting components of $\boldsymbol{Q_2}$, the proposed aromaticity descriptors can be computed using a simple Excel spreadsheet or even a manual calculator.

```
File benzene.fchk Density MP2
Angstrom
Multipoles
  Limit 2
  Switch 4.0
  Radius H 0.35
  Punch file.punch
  Add TOP1 0.0 0.0  1.00
Start
Finish
```

Figure 3S. An input file for the GDMA2 to compute the proposed Q_2-based aromaticity descriptors.

G D M A

by Anthony Stone

Distributed Multipoles from Gaussian wavefunctions

version 2.3.3 (6b8e81e)

Compiled with gfortran on 14 January 2024 at 08:13:23

Starting at 17:23:21 on 31 Jan 2025

Using MP2 density matrix from file benzene.fchk

Distributed Multipole Analysis

Standard DMA for products of primitives with exponent greater than 4.00000

Using 80-point Euler-MacLaurin radial quadrature

Using 590-point Lebedev quadrature

Becke smoothing parameter = 3

Positions and radii in angstrom

Multipole moments in atomic units, ea_0^k for rank k

C x = 0.000000 y = 1.394663 z = -0.000000 angstrom

Maximum rank = 2 Radius = 0.650 angstrom

Q00 = 0.110486

|Q1| = 0.343520 Q10 = 0.231564 Q11s = -0.253741

|Q2| = 1.107127 Q20 = -0.974221 Q21s = -0.427744 Q22c = -0.306038

C x = -1.207814 y = 0.697331 z = -0.000000 angstrom

Maximum rank = 2 Radius = 0.650 angstrom

Q00 = 0.110486

|Q1| = 0.343521 Q10 = 0.231563 Q11c = 0.219748 Q11s = -0.126871

|Q2| = 1.107128 Q20 = -0.974220 Q21c = 0.370440 Q21s = -0.213870

Q22c = 0.153018 Q22s = -0.265042

C x = -1.207814 y = -0.697331 z = -0.000000 angstrom

Maximum rank = 2 Radius = 0.650 angstrom

Q00 = 0.110486

|Q1| = 0.343521 Q10 = 0.231563 Q11c = 0.219748 Q11s = 0.126871

|Q2| = 1.107128 Q20 = -0.974220 Q21c = 0.370440 Q21s = 0.213870

Q22c = 0.153018 Q22s = 0.265042

C x = -0.000000 y = -1.394663 z = -0.000000 angstrom

Maximum rank = 2 Radius = 0.650 angstrom

Q00 = 0.110486

|Q1| = 0.343520 Q10 = 0.231564 Q11s = 0.253741

|Q2| = 1.107127 Q20 = -0.974221 Q21s = 0.427744 Q22c = -0.306038

C x = 1.207814 y = -0.697331 z = -0.000000 angstrom

Maximum rank = 2 Radius = 0.650 angstrom

Q00 = 0.110486

|Q1| = 0.343521 Q10 = 0.231563 Q11c = -0.219748 Q11s = 0.126871

|Q2| = 1.107128 Q20 = -0.974220 Q21c = -0.370440 Q21s = 0.213870

Q22c = 0.153018 Q22s = -0.265042

C x = 1.207814 y = 0.697331 z = -0.000000 angstrom

Maximum rank = 2 Radius = 0.650 angstrom

Q00 = 0.110486

|Q1| = 0.343521 Q10 = 0.231563 Q11c = -0.219748 Q11s = -0.126871

|Q2| = 1.107128 Q20 = -0.974220 Q21c = -0.370440 Q21s = -0.213870

Q22c = 0.153018 Q22s = 0.265042

H x = 0.000000 y = 2.479089 z = -0.000000 angstrom

Maximum rank = 2 Radius = 0.350 angstrom

Q00 = 0.038455

|Q1| = 0.118326 Q10 = 0.007460 Q11s = -0.118091

|Q2| = 0.210686 Q20 = -0.164962 Q21s = -0.004627 Q22c = -0.130977

H x = -2.146954 y = 1.239544 z = -0.000000 angstrom

Maximum rank = 2 Radius = 0.350 angstrom

Q00 = 0.038455

|Q1| = 0.118326 Q10 = 0.007460 Q11c = 0.102269 Q11s = -0.059045

|Q2| = 0.210686 Q20 = -0.164962 Q21c = 0.004007 Q21s = -0.002314

Q22c = 0.065489 Q22s = -0.113430

H x = -2.146954 y = -1.239544 z = -0.000000 angstrom

Maximum rank = 2 Radius = 0.350 angstrom

Q00 = 0.038455

|Q1| = 0.118326 Q10 = 0.007460 Q11c = 0.102269 Q11s = 0.059045

|Q2| = 0.210686 Q20 = -0.164962 Q21c = 0.004007 Q21s = 0.002314

Q22c = 0.065489 Q22s = 0.113430

H x = -0.000000 y = -2.479089 z = -0.000000 angstrom

Maximum rank = 2 Radius = 0.350 angstrom

Q00 = 0.038455

|Q1| = 0.118326 Q10 = 0.007460 Q11s = 0.118091

|Q2| = 0.210686 Q20 = -0.164962 Q21s = 0.004627 Q22c = -0.130977

H x = 2.146954 y = -1.239544 z = -0.000000 angstrom

Maximum rank = 2 Radius = 0.350 angstrom

Q00 = 0.038455

|Q1| = 0.118326 Q10 = 0.007460 Q11c = -0.102269 Q11s = 0.059045

|Q2| = 0.210686 Q20 = -0.164962 Q21c = -0.004007 Q21s = 0.002314

Q22c = 0.065489 Q22s = -0.113430

H x = 2.146954 y = 1.239544 z = -0.000000 angstrom

Maximum rank = 2 Radius = 0.350 angstrom

Q00 = 0.038455

|Q1| = 0.118326 Q10 = 0.007460 Q11c = -0.102269 Q11s = -0.059045

|Q2| = 0.210686 Q20 = -0.164962 Q21c = -0.004007 Q21s = -0.002314

Q22c = 0.065489 Q22s = 0.113430

TOP1 x = 0.000000 y = 0.000000 z = 1.000000 angstrom

Maximum rank = 2 Radius = 0.650 angstrom

Q00 = -0.893646

|Q1| = 0.254606 Q10 = 0.254606

|Q2| = 0.423932 Q20 = 0.423932

Total multipoles referred to origin at

x = 0.000000, y = 0.000000, z = 0.000000 angstrom

Q00 = -0.000001

|Q1| = 0.000002 Q10 = -0.000002

|Q2| = 6.142557 Q20 = -6.142557 Q22c = -0.000038

CPU time used: 0m13.537s Total: 0m13.537s

Finished at 17:23:34 on 31 Jan 2025

Figure 4S. An output file for the GDMA2 to compute the proposed $\boldsymbol{Q}_2$-based aromaticity descriptors. The colored numbers are the tensor components of $\boldsymbol{Q}_2$ for computing the proposed aromatic descriptors.

The GDMA2 input automatization

Although preparing the GDMA2 input file is straightforward, sometimes it may be convenient to use the following Python script to automate the preparation of several calculations. This allows for the quick and efficient creation of specific files for different molecules, ensuring standardization and reducing manual errors in running configuration.

To function correctly, the script must be executed within a directory containing subdirectories for each desired calculation, the names of which match those used to generate the corresponding .fchk files. You can change the desired distances in GDMA2 using the "ADD" command.

```
import os

template_input = """File {molecule}.fchk
Angstrom
Multipoles
  Limit 2
  Switch 4.0
  Radius H 0.35
  Punch file.punch
  ADD TOP1 0.0 0.0  1.00 #You must change these to add more distances if
desired
  ADD TOP-1 0.0 0.0 -1.00
Start
Finish
"""

root_dir = os.getcwd()
for folder in os.listdir(root_dir):
    folder_path = os.path.join(root_dir, folder)
    if os.path.isdir(folder_path):

        molecule_name = folder
        input_file = os.path.join(folder_path, f"gdma_{molecule_name}.input")

        with open(input_file, 'w', newline='\n') as file:
            file.write(template_input.format(molecule=molecule_name))
```

The script for computing the descriptors

The following Python script processes the GDMA2 output files containing the computed DMA quadrupole moment components for a given molecule, extracting the specific values of $|\boldsymbol{Q_2}|$ and $\boldsymbol{Q_{2_{zz}}}$ (Q_{20}) for different atoms and molecular sites.

It is worth noting that the extraction of descriptors relating to the ring atoms ($|Q_2|_{ring\ atoms}$ and $Q_{2_{zz,ring\ atoms}}$) is carried out considering that it is a benzene derivative, containing a 6-membered ring (only carbons). This 6-carbon count is done by the commands "`if "C" in line:`" and "`if len(q2_ring_values) == 6`".

The benzene molecule must be present for the normalization step. Therefore, the code expects that, as for all other molecules, a "C6H6" folder containing the respective GDMA2 output has previously been created.

```
import os
import re
import pandas as pd
import glob

def calculate_q2_ring_atoms(lines, start_index, end_index):
    """
    Function to calculate the sum of |Q2| values for ring atoms.
    Returns the sum of the found values or None if fewer than 6 values are
found.
    """
    q2_ring_values = []
    for i in range(start_index, end_index):
        line = lines[i]
        if "C" in line:  # Only carbon atoms
            # Search for the value of |Q2| after finding "C"
            for j in range(i + 1, len(lines)):
                if "|Q2|" in lines[j]:
                    parts = lines[j].split('=')
                    if len(parts) > 1:
                        q2_value = parts[1].split()[0].strip()  # Extract the
number
                        try:
                            q2_ring_values.append(float(q2_value))
                        except ValueError:
```

```
                            print(f"Error converting |Q2| to float:
{q2_value}")
                    if len(q2_ring_values) == 6:  # Stop after finding 6
values
                        return sum(q2_ring_values)  # Return the sum of
values
    print("Less than 6 |Q2| values found.")
    return None


def calculate_qzz_ring_atoms(lines, start_index, end_index):
    """
    Function to calculate the sum of Qzz=Q20 values for ring atoms.
    Returns the sum of the found values or None if fewer than 6 values are
found.
    """
    qzz_ring_values = []
    for i in range(start_index, end_index):
        line = lines[i]
        if "C" in line:  # Only carbon atoms
            # Search for the value of Qzz = Q20 after finding "C"
            for j in range(i + 1, len(lines)):
                if "Q20" in lines[j]:
                    content_after_qzz = lines[j].split("Q20")[1]  # Capture
everything after "Q20"
                    parts = content_after_qzz.split('=')
                    if len(parts) > 1:
                        qzz_value = parts[1].split()[0].strip()  # Extract
the number
                        try:
                            qzz_ring_values.append(float(qzz_value))
                        except ValueError:
                            print(f"Error converting Qzz = Q20 to float:
{qzz_value}")
                    if len(qzz_ring_values) == 6:  # Stop after finding 6
values
                        return sum(qzz_ring_values)  # Return the sum of
values
    print(f"Less than 6 Qzz = Q20 values found.")
```

```
    return None


def calculate_q2_origin(lines):
    """
    Function to calculate the |Q2| value for the origin.
    Returns the value found or None if no value is found.
    """
    q2_origin = None
    for i, line in enumerate(lines):
        if 'Total multipoles referred to origin' in line:
            # Search for the values after the line 'Total multipoles referred
to origin'
            for j in range(i + 1, len(lines)):
                if '|Q2|' in lines[j]:
                    parts = lines[j].split('=')
                    if len(parts) > 1:
                        q2_origin = parts[1].split()[0].strip()  # Extract
the number
                        try:
                            q2_origin = float(q2_origin)
                        except ValueError:
                            print(f"Error converting |Q2| origin to float:
{q2_origin}")
                    break
            break

    if q2_origin is None:
        print("No |Q2| origin value found.")
    return q2_origin

def calculate_qzz_origin(lines):
    """
    Function to calculate the Qzz (Q20) value for the origin.
    Returns the value found or None if no value is found.
    """
    qzz_origin = None
    for i, line in enumerate(lines):
        if 'Total multipoles referred to origin' in line:
```

```
            # Search for the values after the line 'Total multipoles referred
to origin'
            for j in range(i + 1, len(lines)):
                if 'Q20' in lines[j]:
                    content_after_qzz = lines[j].split("Q20")[1]  # Capture
everything after "Q20"
                    parts = content_after_qzz.split('=')
                    if len(parts) > 1:
                        qzz_value = parts[1].split()[0].strip()  # Extract
the number
                        try:
                            qzz_origin = float(qzz_value)
                        except ValueError:
                            print(f"Error converting Qzz origin to float:
{qzz_value}")
                    break
            break

    if qzz_origin is None:
        print("No Qzz origin value found.")
    return qzz_origin


def calculate_tops(lines):
    """
    Function to calculate the values of |Q2|(n) and Q2(n)zz for all TOPs
found.
    Returns a dictionary with the found values.
    """
    tops_data = {}
    for i, line in enumerate(lines):
        # Use regex to find TOP(n) or TOPn, where n can be a number or a
letter
        match = re.search(r"TOP(?:\(([\w\-]+)\)|([\w\-]+))", line)
        if match:
            # Extract the TOP identifier (first group captures TOP(n), second
group captures TOPn)
            top_id = match.group(1) or match.group(2)
```

```
            # Search for the |Q2| and Q20 values in the following lines
            q2_value = None
            q20_value = None
            for j in range(i + 1, i + 7):  # Considers up to 6 lines after
the TOP line
                if j >= len(lines):
                    break

                # Search for |Q2|
                if '|Q2|' in lines[j]:
                    content_after_q2 = lines[j].split('|Q2|')[1]  # Capture
everything after '|Q2|'
                    parts = content_after_q2.split('=')
                    if len(parts) > 1:
                        q2_str = parts[1].split()[0].strip()  # Extract the
numeric value
                        try:
                            q2_value = float(q2_str)
                        except ValueError:
                            print(f"Error converting |Q2| to float in
TOP({top_id}): {q2_str}")


                # Search for Q20
                if 'Q20' in lines[j]:
                    content_after_q20 = lines[j].split('Q20')[1]  # Capture
everything after 'Q20'
                    parts = content_after_q20.split('=')
                    if len(parts) > 1:
                        q20_str = parts[1].split()[0].strip()  # Extract the
numeric value
                        try:
                            q20_value = float(q20_str)
                        except ValueError:
                            print(f"Error converting Q20 to float in
TOP({top_id}): {q20_str}")


            # Store the values in the dictionary
```

```
            if q2_value is not None and q20_value is not None:
                tops_data[top_id] = {"|Q2|": q2_value, "Q20": q20_value}

    return tops_data


def process_output_file(output_file, folder_name):
    try:
        with open(output_file, 'r') as file:
            lines = file.readlines()

        start_marker = 'Multipole moments in atomic units, ea_0^k for rank k'
        end_marker = 'CPU time used'

        # Find the positions of the markers
        start_index = next((i + 2 for i, line in enumerate(lines) if
start_marker in line), None)
        end_index = next((i for i in range(start_index, len(lines)) if
end_marker in lines[i]), None) if start_index else None

        if start_index is None or end_index is None:
            print(f"Error processing {output_file}: Markers not found
correctly.")
            return

        # Calculate values
        total_q2_ring = calculate_q2_ring_atoms(lines, start_index,
end_index)
        total_qzz_ring = calculate_qzz_ring_atoms(lines, start_index,
end_index)
        total_q2_origin = calculate_q2_origin(lines)
        total_qzz_origin = calculate_qzz_origin(lines)
        tops_data = calculate_tops(lines)

        # Create the result dictionary
        result = {
            'Molecule': folder_name,
            'Total |Q2| (ring atoms)': total_q2_ring,
            'Total Qzz (ring atoms)': total_qzz_ring,
```

```python
            '|Q2| origin': total_q2_origin,
            'Qzz origin': total_qzz_origin,
        }

        if tops_data:
            for top_id, values in tops_data.items():
                result[f'|Q2|({top_id})'] = values['|Q2|']
                result[f'Q2({top_id})zz'] = values['Q20']
        else:
            print('No additional sites specified')

        # Display results
        print(f"Results for folder '{folder_name}':")
        for label, value in result.items():
            print(f"{label}: {value}")

        print("-" * 50 + "\n")

        return result

    except Exception as e:
        print(f"Error processing {output_file}: {e}")
        return None


def save_to_csv(results, csv_filename):
    df = pd.DataFrame(results)
    df.to_csv(csv_filename, index=False)
    print(f"Results saved to: {csv_filename}")

def normalizer(df):
    # Create a copy of the dataframe to store normalized values
    df_normalized = df.copy()

    # Loop through the columns (excluding 'Molecule') to normalize each one
    for column in df.columns[1:]:  # Skip the 'Molecule' column
        # Get the value of C6H6 for the current column
        if 'C6H6' in df['Molecule'].values:
            benzene_value = df[df['Molecule'] == 'C6H6'][column].values[0]
```

```
            # Normalize the column values (divide by benzene value and
multiply by 100)
            df_normalized[column] = df[column] / benzene_value

    # Save the normalized results to a new CSV file
    df_normalized.to_csv('normalized_results.csv', index=False)
    print("Normalized results saved to 'normalized_results.csv'.")

# Main function
def main():
    root_directory = "."  # Root directory
    all_results = []  # List to store the results

    # Traverse all folders in the root directory
    for folder_name in os.listdir(root_directory):
        folder_path = os.path.join(root_directory, folder_name)

        if os.path.isdir(folder_path):
            output_files = glob.glob(os.path.join(folder_path,
"*gdma*.output"))

            if output_files:
                for output_file in output_files:
                    result = process_output_file(output_file, folder_name)

                    # If results are found, add them to the list
                    if result:
                        all_results.append(result)
            else:
                print(f"No 'gdma*.output' file found in folder:
{folder_path}")

    if all_results:
        # Save the results to a CSV
        save_to_csv(all_results, "output_results.csv")
        dataframe = pd.DataFrame(all_results)
```

```
        normalizer(dataframe)
    else:
        print("No results found to save.")


if __name__ == "__main__":
    main()
```

References

(1) Guest, M. F.; Bush, I. J.; Van Dam, H. J. J.; Sherwood, P.; Thomas, J. M. H.; Van Lenthe, J. H.; Havenith, R. W. A.; Kendrick, J. The GAMESS-UK electronic structure package: algorithms, developments and applications. *Mol. Phys.* **2005**, *103* (6-8), 719-747. DOI: 10.1080/00268970512331340592.

(2) Barca, G. M. J.; Bertoni, C.; Carrington, L.; Datta, D.; De Silva, N.; Deustua, J. E.; Fedorov, D. G.; Gour, J. R.; Gunina, A. O.; Guidez, E.; et al. Recent developments in the general atomic and molecular electronic structure system. *J. Chem. Phys.* **2020**, *152* (15). DOI: 10.1063/5.0005188 (acccessed 12/16/2024).

(3) Werner, H.-J.; Knowles, P. J.; Knizia, G.; Manby, F. R.; Schütz, M. Molpro: a general-purpose quantum chemistry program package. *WIREs Comp. Mol. Sci.* **2012**, *2* (2), 242-253. DOI: https://doi.org/10.1002/wcms.82.

(4) Epifanovsky, E.; Gilbert, A. T. B.; Feng, X.; Lee, J.; Mao, Y.; Mardirossian, N.; Pokhilko, P.; White, A. F.; Coons, M. P.; Dempwolff, A. L.; et al. Software for the frontiers of quantum chemistry: An overview of developments in the Q-Chem 5 package. *J. Chem. Phys.* **2021**, *155* (8), 084801. DOI: 10.1063/5.0055522 From NLM.

(5) Smith, D. G. A.; Burns, L. A.; Simmonett, A. C.; Parrish, R. M.; Schieber, M. C.; Galvelis, R.; Kraus, P.; Kruse, H.; Di Remigio, R.; Alenaizan, A.; et al. PSI4 1.4: Open-source software for high-throughput quantum chemistry. *J. Chem. Phys.* **2020**, *152* (18). DOI: 10.1063/5.0006002 (acccessed 12/16/2024).

(6) Amos, R. D.; Rice, J. E. Implementation of analytic derivative methods in quantum chemistry. *Computer Physics Reports* **1989**, *10* (4), 147-187. DOI: https://doi.org/10.1016/0167-7977(89)90001-4.

(7) Stone, A. J. Distributed multipole analysis: Stability for large basis sets. *J. Chem. Theory Comput.* **2005**, *1* (6), 1128-1132, Article. DOI: 10.1021/ct050190+.
